# Design of Antigen-Targeting Fluorogenic Probes Utilizing Intramolecular Addition Reaction of Protein-Dye Hybrids

**DOI:** 10.1021/jacs.5c04193

**Published:** 2025-07-23

**Authors:** Mamiko Nakadate, Ryosuke Kojima, Naoki Seike, Ryo Tachibana, Kyohhei Fujita, Reiko Tsuchiya, Mako Kamiya, Andreas Plückthun, Yasuteru Urano

**Affiliations:** † Graduate School of Medicine, 13143 The University of Tokyo, Tokyo 113-0033, Japan; ‡ Graduate School of Pharmaceutical Sciences, The University of Tokyo, Tokyo 113-0033, Japan; § Laboratory for Chemistry and Life Science, Institute of Integrated Research, Institute of Science Tokyo, Kanagawa 226-8501, Japan; ∥ Department of Biochemistry, 27217 University of Zurich, Zurich 8057, Switzerland

## Abstract

Fluorogenic probes for antigens are useful for various purposes, including medical diagnostics and imaging, but achieving a rapid, large fluorescence increase is difficult. Here, we report a new class of fluorogenic probes for antigens based on a conjugate of an antibody-mimetic DARPin bearing a site-specifically incorporated cysteine and silicon-pyronine (SiP), which reacts reversibly with thiols. By using a library-screening approach, we found that the fluorescence of SiP conjugated to a DARPin is quenched via π-deconjugating addition reaction of the cysteine installed in the DARPin to SiP. Upon antigen binding, the equilibrium of this reaction is shifted to dissociation, restoring π-conjugation in the SiP and resulting in a large increase in fluorescence. As proof of concept of this chemical design principle, we constructed fluorogenic probes targeting GFP and EpCAM, which showed 25- and 12-fold fluorescence increases upon binding, respectively. The latter probe enabled wash-free cancer cell imaging with a low background.

## Graphical abstract

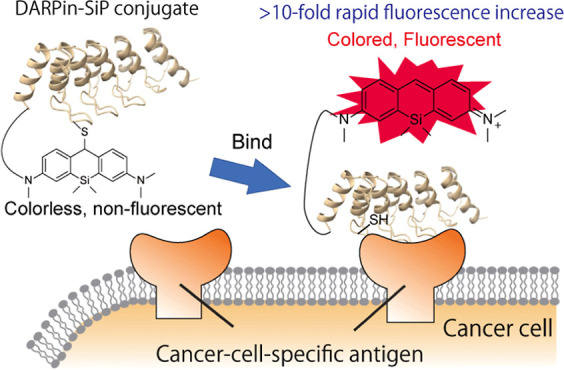

## Introduction

Activatable fluorescence probes (fluorogenic probes) that emit a signal only in the presence of a target biomolecule are useful for various purposes, including fluorescence-guided cancer surgery.^1–3^ For example, several researchers including us have developed fluorogenic probes for cancer-specific enzymes to rapidly visualize cancer cells with low background.^1–11^ However, it is not always possible to find an appropriate biomarker enzyme, depending on the cell types to be visualized. To solve this issue, fluorogenic probes that can directly sense the presence of antigens would be useful to leverage cancer-specific biomarkers without enzymatic activities.^12–14^ However, while many antibody-fluorophore conjugates have been reported,^15,16^ it is difficult to design good fluorogenic probes for antigens, because it is chemically not easy to transmit the information that “the antibody has bound to the antigen” to the fluorophore conjugated to the antibody. There are only a few pioneering examples, including an antibody-pH probe conjugate that senses the acidic intracellular organelle environment after antigen internalization,^17^ and Quenchbodies^18–23^ for direct fluorogenic sensing of antigen. However, the former involves a prolonged delay after probe administration until antigen internalization occurs, and the latter often generates only a small fluorescence increase due to the incomplete control of fluorescence.

Here, we report a new class of fluorogenic probes that directly and rapidly detect the presence of antigens based on a unique chemical mechanism. Specifically, we designed an antigen-targeting fluorogenic probe by conjugating silicon-pyronine (SiP) to an antibody-mimetic, a designed ankyrin repeat protein (DARPin)^24^ bearing a site-specifically incorporated cysteine. The intramolecular π-deconjugating addition reaction of the thiol in the Cys side chain incorporated into the DARPin to SiP achieves strong initial quenching of fluorescence, and the rapid shift of the equilibrium of this reversible addition reaction to the dissociated state (restoring the π-conjugation of SiP) upon antigen binding leads to a very rapid increase of fluorescence. As proof of concept we designed and synthesized probes directed against a model antigen, enhanced green fluorescent protein (GFP), and a cancer-specific antigen, epithelial cell adhesion molecule (EpCAM). These probes showed 25- and 12-fold fluorescence increases, respectively, upon binding to the target. Notably, the EpCAM-targeting probe enabled wash-free imaging of cancer cells expressing EpCAM with a low background.

## Results

### Design of a New Class of Fluorogenic Probes for Antigens

A fluorophore absorbs excitation light, forming a singlet excited state, and fluorescence is emitted during the relaxation process from the excited state. Therefore, OFF/ON switching of fluorescence can be achieved by controlling either the absorption of excitation light or the quantum yield (QY) of fluorescence during the relaxation process.^3^ (For simplicity, we do not discuss probes based on wavelength shift here; see Figure S1 for details).

Many fluorogenic probes rely on the change of QY of fluorescence between the OFF state and ON state. This is often achieved by appropriately controlling the competition between the fluorescence process and nonradiative relaxation processes by means of photoinduced electron transfer (PeT) or Förster resonance energy transfer (FRET), so that the QY drastically increases upon sensing the target molecule (hereafter called “QY-dependent” probes). Many calcium fluorescence probes (e.g., Fluo-3 ^25^) are typical QY-dependent fluorogenic probes, and Quenchbodies^18–23^ for fluorogenic sensing of antigens are also basically QY-dependent. However, although the QY of Quenchbodies is controlled by PeT from tryptophan near the paratope or by dye–dye interaction, it is difficult to achieve sufficient quenching of fluorescence before antigen binding, which results in a relatively low fold increase of fluorescence upon sensing the antigen (typically around 1.5–5 fold for Quenchbodies,^18–23^ though with some exceptions depending on the target^21^).

On the other hand, several researchers including us have reported fluorogenic probes based on control of the absorption of the fluorophore (hereafter called “absorption-dependent” probes).^5,26–31^ For example, we have developed a series of absorption-dependent fluorogenic probes by optimizing the intramolecular spirocyclization strategy to sense biomolecules such as hypochlorite (probe: HySOx^26^), γ-glutamyl transferase (probe: gGlu-HMRG^5,27^ ), and β-galactosidase (probe: HMRef-β-Gal^28^). While the OFF state of QY-dependent probes generally consists of a homogeneous population of colored molecules showing low QY (but often not perfectly quenched), the OFF state of the absorption-dependent fluorogenic probes generally consists of a mixture of two forms in equilibrium: molecules that are completely colorless/nonfluorescent due to π-deconjugation, and π-conjugated colored/fluorescent molecules (Figure S1). Therefore, if the equilibrium can be strongly biased so that the molecules in the π-deconjugated colorless state dominate in the absence of the target molecules and the π-conjugated colored molecules dominate after sensing the target molecules, the absorption-dependent fluorogenic probes can show a much larger fold increase of fluorescence than typical QY-dependent fluorogenic probes. For example, gGlu-HMRG and HMRef-β-Gal indeed show fluorescence increases of >400 fold and >1400 fold, respectively, upon irreversible reaction with their target enzyme,^27,28^ which are remarkably larger increases than those of typical QY-based probes (of the order of 10-fold).

We therefore decided to focus on absorption-dependent fluorogenic probes in order to sense antigens. Since no general design principles are available for such antigen probes, we set out in this study to explore a new design strategy to obtain a dynamic change of fluorescence of such absorption-dependent fluorogenic probes in response to target antigens.

For this purpose, we focused on a far-red to near-infrared emitting fluorophore, SiP650, that we had previously found to react reversibly with thiols (hereafter called SiP, see also Figure S1).^32^ We hypothesized that by site-specifically incorporating a cysteine (Cys) residue into an appropriate position of an antigen-binding protein so that it can react with conjugated SiP, it would be possible to switch SiP to a colorless and nonfluorescent state through π-deconjugation of SiP by the thiol in the absence of antigen (Figure 1a). Then, considering that the addition reaction of a thiol to SiP is reversible,^32^ we anticipated that, if the binding of the antigen to the antigen-binding protein sterically hinders the Cys residue or induces structural distortion, the equilibrium of the SiP-thiol reaction might be shifted toward the dissociated state, thereby restoring both the absorption and fluorescence of SiP. To test this concept, we decided to use a DARPin^24^ as the antigen-binding protein, since it has favorable characteristics for engineering purposes, being small, stable, and easy-to-express. Further, unlike other typical antigen-binding proteins (e.g., antibodies, scFv, nanobodies), DARPins generally do not contain cysteines and thus their function is independent of Cys. We considered this to be useful for precise control of the reaction of the thiol with SiP, because we can focus only on the reaction of Cys installed by point mutation at a specified site.

**Figure 1:**
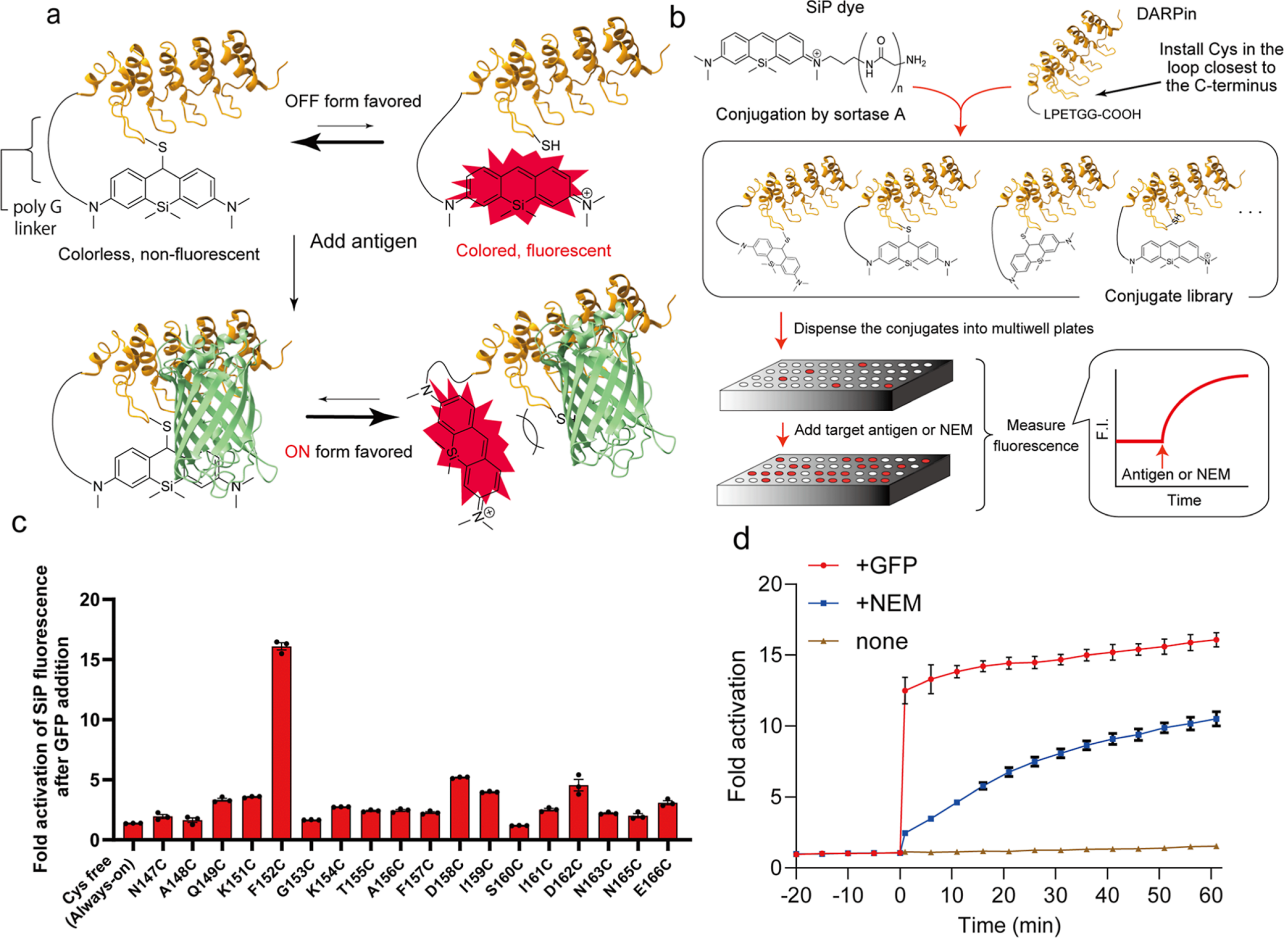
Proof of concept of an antigen-targeting fluorogenic probe based on the DARPin-SiP conjugate using GFP as a model antigen. (a) Schematic illustration of the probe design. In the absence of the target antigen, SiP is π-deconjugated by reaction with the thiol in the side chain of the site-specifically installed Cys in the DARPin (OFF state). We expected that the equilibrium of the reaction of SiP and thiol could be shifted to the dissociated state in the presence of the target antigen when the conjugate is appropriately designed. The dissociation of SiP and thiol leads to recovery of the π-conjugation of SiP, resulting in an increase of both absorption and fluorescence (ON state). (b) Schematic illustration of the screening of DARPin-SiP conjugates. SiP and DARPin mutants were conjugated by a sortase-mediated reaction, and the change of fluorescence upon addition of the antigen was monitored by a multiwell plate reader. In parallel, we monitored the fluorescence increase of the conjugates after treatment with *N*-ethylmaleimide (NEM) in order to evaluate how efficiently the initial fluorescence is quenched. (c) Fold activation of SiP fluorescence of each 5-Gly-SiP conjugate upon GFP addition (the higher, the better). The values of fold activation are calculated by dividing the fluorescence intensity at 61 min by the fluorescence intensity just before the addition of GFP. (d) Time course of the fold change of fluorescence of the hit probe: the conjugate of 3G86.32 F152C and 5-Gly SiP. GFP or NEM was added after the measurement at 0 min. For (c,d), conditions were as follows: Conjugate: 500 nM, GFP: 1 μM, NEM: 1 mM. Error bars represent ± SEM (*n* = 3). Fluorescence was measured with an Envision plate reader. Excitation: 620 nm (±5 nm), Emission: 685 nm (±17.5 nm).

### Proof of Concept of Our Absorption-Dependent Antigen Probe Design by Using GFP as a Model Antigen

For proof of concept of our probe design, we selected GFP as a first model target (we used the anti-GFP DARPin 3G86.32 ^33^ C76N as a template, hereafter called 3G86.32). Having decided to take a screening approach using a library of DARPin-SiP conjugates (Figure 1b), we first prepared a series of plasmids encoding 3G86.32 bearing a sortase A recognition sequence^34^ at its C-terminus and a Cys substitution in or around the antigen-recognizing loop sequence of the DARPin (the loop closest to the C-terminus). In parallel, we prepared derivatives of SiP bearing 5- or 6-residue poly glycine for sortase-mediated conjugation (hereafter 5Gly-SiP and 6Gly-SiP, respectively) (Schemes S1–S8). After conjugating the SiP derivatives to the purified DARPins by using sortase A (Figure S2), we monitored the change in fluorescence of SiP following the addition of an excess of GFP (Figure 1b). The fluorescence increase of SiP in the conjugates treated with *N*-ethylmaleimide (NEM)^35^ was also measured in order to evaluate how efficiently the initial fluorescence is quenched and what the maximum fluorescence of each conjugate might be, based on the assumption that NEM gradually traps the thiol of the SiP-DARPin conjugate when SiP is dissociated from the thiol, finally shifting the equilibrium of the thiol/SiP reaction completely to the π-conjugated ON state.

As a result, we found the fluorescence of SiP is well quenched in many of the conjugates of DARPin and 5Gly-SiP (Figures S3a and S4). Moreover, the conjugate of F152C mutant and 5Gly-SiP worked as a good GFP-activatable probe, showing a 16-fold activation of SiP fluorescence upon the addition of GFP (Figure 1c). Since this fluorescence fold activation after GFP addition is even higher than that after NEM addition (Figure S3b), it seems likely that the quenching of the probe is fully unlocked. It is particularly interesting that the activation of SiP fluorescence occurred essentially immediately after the addition of GFP, while the reaction with NEM is not complete within this time scale (Figure 1d, see Discussion section and for interpretation of this result). The screening results with 6Gly-SiP were similar to those with 5Gly-SiP, suggesting that the screening works reliably with different probes, and the length of the flexible linker does not dramatically affect the results, at least in this setting (Figure S5). Given the higher fold activation of fluorescence of F152C-5Gly-SiP conjugate, we used this conjugate for further assays. Hereafter, we refer to 3G86.32­(F152C)-5Gly-SiP conjugate as the “activatable GFP probe” and 3G86.32­(no Cys)-5Gly-SiP as the “always-on GFP probe”.

Next, we compared both the absorption and fluorescence spectra of SiP of the activatable and always-on GFP probes before and after the addition of GFP (Figures 2a,b, S6). As a result, a drastic increase in both absorption and fluorescence of SiP was observed in the case of the activatable probe, but not the always-on probe. The increase of absorption indicates that the SiP is initially π-deconjugated by the side-chain thiol of the incorporated Cys, and the π-conjugation is restored after the addition of GFP (i.e., the probe is absorption-dependent, as expected.). The similar values of fluorescence QY of SiP regardless of the presence of GFP (target antigen) or of cysteine in the DARPin (Figure 2c) also indicate that the change of fluorescence of the activatable probe is mostly due to the change in the OFF/ON status of absorbance of SiP (wavelength shift was not observed at least under the tested conditions, as shown in Figure S6b), which further confirms that the probe works as designed. The absorbance and fluorescence intensities of equal concentrations of the activatable and always-on probes after the addition of the GFP were almost the same, indicating that the equilibrium of the SiP-thiol reaction was almost completely shifted to the ON state by the addition of GFP. Together with the fact that the QY values of the SiP conjugates are comparable to that of free SiP (0.39 ^32^) and the reasonable level of SiP absorbance after GFP addition relative to GFP’s own absorbance (Figure S6a), these results suggest that the brightness of the probe after sensing the target antigen is close to the theoretical maximum.

**Figure 2:**
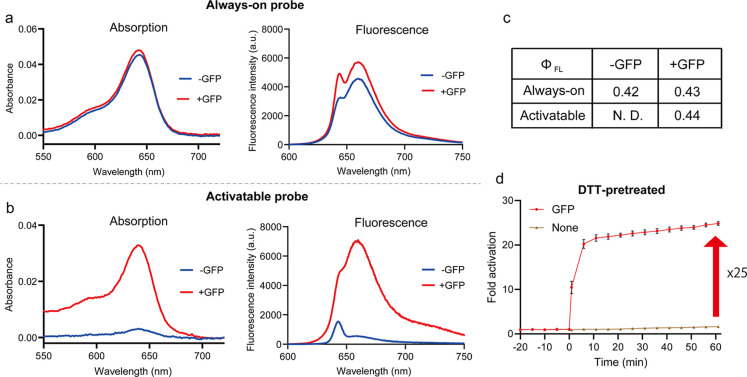
Detailed characterization of the always-on GFP probe (3G86.32–5Gly-SiP) and the activatable GFP probe (3G86.32 F152C-5Gly-SiP). All the data in this figure was measured in 10 mM HEPES buffer (pH 7.4). (a) Absorption and fluorescence spectra (in the wavelength region where SiP has its main absorption) of the always-on probe (500 nM) with or without GFP (1 μM). (b) Absorption and fluorescence spectra of the activatable probe (500 nM) with or without GFP (1 μM). Excitation for fluorescence: 643 nm. See Figure S6 for full spectrum including GFP absorption. (c) Fluorescence quantum yields of the always-on and activatable GFP probes. N.D.: not determined due to the low absorbance. Probe: 500 nM, GFP: 1 μM. Excitation: 643 nm. (d) Time course of the fold change of SiP fluorescence of the activatable GFP probe pretreated with DTT. After the pretreatment, DTT was removed before use by changing the buffer to recover intact SiP that can reversibly react with DTT. Probe: 500 nM, GFP: 1 μM (the same as for Figure 1c,d). Error bars represent ± SEM (*n* = 3).

In principle, if the probe forms dimers involving the incorporated Cys (i.e., forming a disulfide bond), this would lead to an unwanted increase of the background fluorescence, because the SiP cannot be π-deconjugated. Therefore, to further improve the performance of the activatable probe, we pretreated the probe with dithiothreitol (DTT) to reduce the S–S form. Indeed, we found the fold activation of fluorescence after the addition of GFP then reached 25-fold with this pretreatment (Figure 2d). Finally, the *K*_d_ of the probe for GFP was calculated to be 96 nM, based on the fluorescence increase (Figure S7). This value is larger than that of the original DARPin (*K*_d:GFP_ of 3G86.32: < 5 nM measured using fluorescence anisotropy^33^); however, despite this difference we consider that the result is acceptable for proof of concept of the probe design.

### Development of a Fluorogenic Probe for the Cancer-Specific Antigen EpCAM

Following the success of the GFP probe, we next set out to develop a fluorogenic probe for EpCAM, a surface biomarker of certain types of cancer cells^36–38^ (Figure 3a). We adopted the DARPin Ec1 ^39^ as the anti-EpCAM DARPin, and took a similar screening approach to that in the case of the GFP probe. By measuring the change of SiP fluorescence of the conjugates of Ec1 Cys mutants and 5-Gly SiP following the addition of the extracellular domain of EpCAM (EpCAM ECD), we identified several conjugates (K152C, L157C, N161C) that exhibited significant fluorescence increases (Figures 3b, S8 and S9) (many of the conjugates were initially quenched, similar to what we observed with the GFP probe (Figure S8a)). Among the candidates, we selected the K152C conjugate that shows a 7-fold fluorescence increase within a few minutes (Figure S9) for further analysis. This conjugate was particularly promising because the fold activation observed after EpCAM ECD addition was comparable to that observed after NEM treatment, suggesting that the fluorescence quenching was fully relieved upon antigen recognition (Figure S8b). The fold activation of fluorescence after the addition of EpCAM ECD reached more than 12-fold when DTT pretreatment was applied (Figure 3c). The *K*_d_ of the probe for EpCAM ECD was calculated to be 238 nM, based on the fluorescence increase (Figure S10). Again, though this value is higher than that of the original DARPin (*K*_d_:EpCAM of Ec1:68 pM, measured by surface plasmon resonance^39^), we think it is acceptable for an imaging agent. Hereafter, we refer to the Ec1-5Gly-SiP conjugate as the “activatable EpCAM probe” and Ec1­(no Cys)-5Gly-SiP as the “always-on EpCAM probe”.

**Figure 3:**
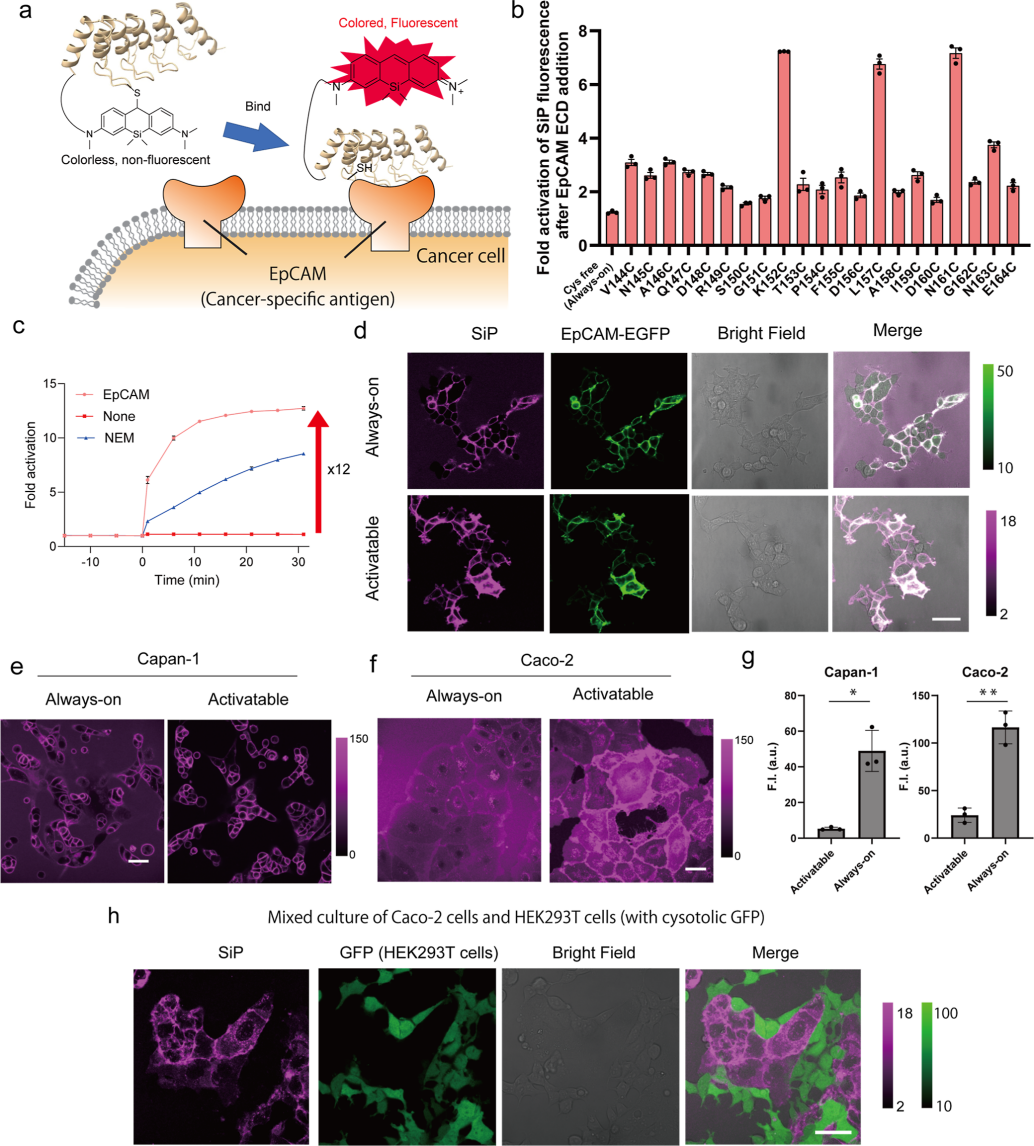
Development of a fluorescent probe for detecting the cancer-specific cell–surface antigen EpCAM. (a) Schematic illustration of the probe design. (b) Fold activation of SiP fluorescence of each conjugate upon the addition of the extracellular domain of EpCAM (EpCAM ECD). Conjugate: 500 nM (not pretreated with DTT), EpCAM ECD: 1 μM. The values of fold activation were calculated by dividing the fluorescence intensity at 31 min by that just before the addition of EpCAM ECD (0 min). Error bars represent ± SEM (*n* = 3). (c) Time course of fold change of fluorescence intensity of the activatable EpCAM probe (Ec1 K152C-5-Gly SiP conjugate) following the addition of EpCAM ECD or NEM. The probe was pretreated with DTT. Probe: 500 nM, EpCAM ECD or NEM: 1 μM or 1 mM, respectively. Error bars represent ± SEM (*n* = 3). (d) Live-cell imaging of HEK293T cells expressing EpCAM-EGFP without washing. (e) Live-cell imaging of Capan-1 cells without washing. (f) Live-cell imaging of Caco-2 cells without washing. (g) Quantification of background fluorescence for (e,f). Two additional fields of view (Figure S13) were simultaneously analyzed, and average background fluorescence in each image was used as the individual data point. Error bars: ± SD (*n* = 3). *p < 0.05, **p < 0.01; two-tailed Welch’s *t*-test. (h) Live-cell imaging of mixed coculture of EpCAM-positive Caco-2 cells and EpCAM-negative HEK293T cells (expressing cytosolic GFP as a marker) with the activatable probe. For (d–h), 500 nM of activatable EpCAM in PBS was used. The images were obtained with a Leica TCS SP5 within 30 min after the addition of the probe, with a maximum pinhole size to emphasize the background (see Figure S12 for this effect). Ex: 633 nm, Em: 653–750 nm for SiP fluorescence (shown in magenta), and Ex: 488 nm, Em: 500–600 nm for GFP fluorescence. Scale bars: 50 μm. Note that look-up tables cannot be directly compared between each subfigure, due to the difference of microscope settings (e.g., laser intensity, detection sensitivity).

We next applied the EpCAM probes for live cell imaging of HEK293T cells expressing EpCAM fused to the cytoplasmic tail of EGFP (EpCAM-EGFP). Expression of EpCAM itself in the original HEK293T cells is negligible (Figure S11). SiP fluorescence was selectively observed from the surface of the cells expressing EpCAM-EGFP, and the GFP fluorescence and SiP fluorescence were well correlated, indicating the specificity of the probe for EpCAM (Figures 3d and S12a). Gratifyingly, we confirmed a lower fluorescence signal from the area without cells when the activatable probe was applied (note that the pinhole of the confocal microscope was maximized to make the background signal easily visible, which explains why the fluorescence signal appears to be partially emitted from the intracellular region (Figure S12b)). In contrast, washing was necessary to remove the background signal when the always-on probe was used (Figure S12c), establishing that activatability is secured under the conditions of live-cell imaging. We also confirmed that the antigen binding of the activatable probe is sufficiently strong to withstand the washing process (Figure S13c). Throughout the observation, the brightness levels of the always-on probe and activatable probe at the cell membrane were comparable, again suggesting that the quenching of the activatable probe is fully relieved upon antigen recognition. We also confirmed that the probe is functional in multiple cell-culture media including HBSS, OptiMEM, and DMEM with 10% FBS (Figure S12d).

Next, we applied the probes to Capan-1 cells (human pancreatic adenocarcinoma) and Caco-2 cells (human colorectal adenocarcinoma), both of which express high levels of EpCAM^36,40^ (Figure S11). Again, the activatable EpCAM probe showed very low background fluorescence, enabling strikingly vivid imaging of the EpCAM-positive cancer cells (Figure 3e,f,g; see Figure S13a,b for the bright-field images and additional images used for signal quantification). Visualization was achieved within a few minutes after the addition of the probes without washing (Figure S13c), which is only possible with an activatable probe that directly senses the binding of the target antigen. Further, by using the activatable EpCAM probe, we confirmed that Capan-1 and Caco2 can be selectively visualized when cocultured with EpCAM-negative HEK293T cells without washing, highlighting the usefulness of the developed probe in the mixed population of EpCAM-positive and negative cells (Figures 3h and S13d).

### Structural Modeling and Analysis of Actuation Mechanism of the Fluorogenic Antigen Probes

To further support our findings, we conducted structural analysis of the developed DARPin–SiP conjugates. Taking into consideration the crystal structure of the complex of GFP and the anti-GFP DARPin,^41^ we conducted structural calculation of the anti-GFP conjugates, K151C, F152C (hit activatable probe), and G153C by a quantum mechanics/molecular mechanics (QM/MM) method (Figure 4, Data S1–S12). Specifically, we compared the energy values of 4 states per complex: OFF state (SiP π-deconjugated) without GFP, OFF state with GFP, ON state (SiP π-conjugated), and ON state with GFP (Figure 4a). These calculations suggest that Cys reacts readily with SiP in the absence of GFP, and the OFF state is generally more stable than the ON state in the absence of GFP (Figure 4b).

**Figure 4:**
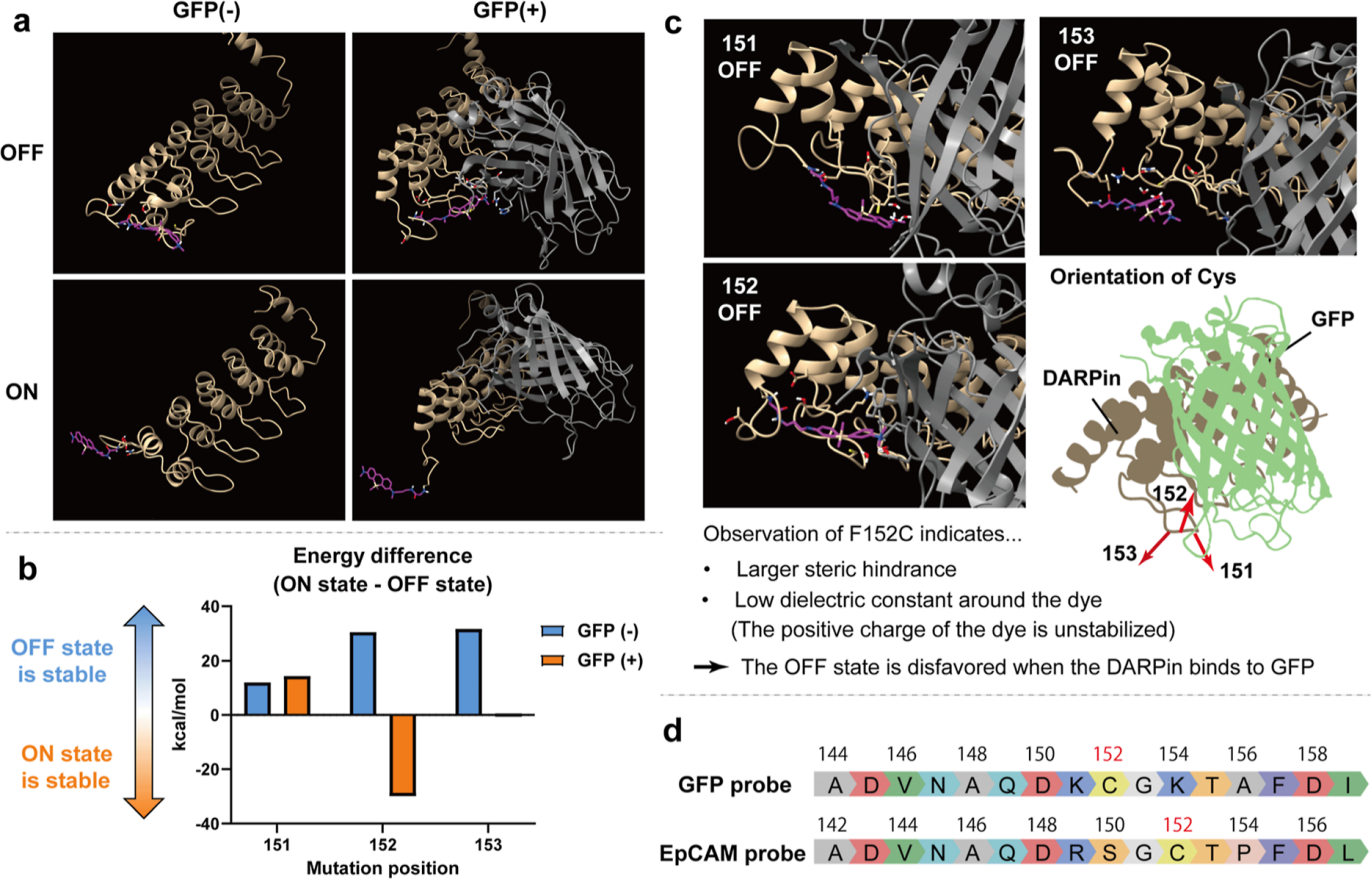
Structural modeling and analysis of the developed activatable probes. (a) Schematic of the calculations for the activatable GFP probe. Energy values of the 4 states (OFF/ON state without/with the target antigen GFP) were calculated by a QM/MM, F152C is shown as an example. DARPin, SiP, and GFP are shown in pale yellow, pink, and gray, respectively. (b) Difference of the QM/MM energy between OFF state and ON state (with or without GFP) of the K151C, F152C, and G153C conjugates. Without GFP, the OFF state was calculated to be generally favored. When each conjugate binds to GFP, the ON state is favored only in the case of the F152C conjugate, which is consistent with the experimental results. (c) Close-up of the OFF state of each conjugate with GFP. Larger steric hindrance and low dielectric constant around the dye were observed only in the case of F152C conjugate; this should destabilize the OFF state with GFP. (d) Comparison of the best Cys positions between the activatable GFP probe and activatable EpCAM probe.

This is consistent with the fact that many of the conjugates are initially quenched (Figure S3a). Furthermore, it appeared the ON state is stabilized in the presence of GFP only in the case of the F152C mutant (hit probe) (Figure 4b). The consistency of the experimental results and calculations further confirms that the probe functioned as designed. Close observation of the OFF state of each conjugate with GFP suggested that larger steric hindrance and low dielectric constant around the dye (which should make positively charged SiP unstable) exist only in the case of F152C conjugate, suggesting that these are the driving forces that disfavor the OFF state of F152C conjugate in the presence of GFP (Figure 4c). Although we could not conduct detailed structural calculations for the EpCAM probe because the crystal structure of the complex of EpCAM and anti-EpCAM DARPin is unavailable, the position of Cys in the activatable EpCAM probe is close to that in the activatable GFP probe (Figure 4d) suggesting that activation mechanisms of the two probes are similar. Indeed, we confirmed that treatment of both GFP probe and EpCAM probe with high concentrations of guanidine, which is known to disrupt protein structure, resulted in some fluorescence increase (Figure S14), suggesting that the quenching modes of the two probes are similar from the viewpoint that the steric structure is key. Here, it should be noted that the overlap in hit residue numbering is coincidental: as shown in Figure 4d, when the sequences of the GFP-binding DARPin and the EpCAM-binding DARPin are aligned, there is a two-residue offset between them. Furthermore, since L157C and N161C also functioned as activatable EpCAM probes (Figure 3b), antigen-specific factor(s) must be involved as well.

## Discussion

We have designed and synthesized protein-dye hybrid fluorogenic probes that show a large, very rapid (within tens of seconds) fluorescence increase in the presence of target antigens by applying the intramolecular nucleophilic addition reaction previously used to construct small-molecule-based absorption-dependent fluorogenic probes. Specifically, we found that precise control of the reversible addition reaction of the thiol of Cys incorporated in antibody-mimetic DARPins to the thiol-reactive fluorophore SiP enabled a remarkable fold activation of both absorption and fluorescence upon antigen binding. To our knowledge, the probes developed in this study are the first absorption-dependent antigen-sensing probes that show dramatic OFF/ON switching (>10 fold) of the fluorescence signal, providing a larger fold fluorescence increase than typical QY-dependent Quenchbodies (several fold). Thus, this work opens up a new chemical strategy to build antigen-targeting fluorogenic probes.

In this study, we demonstrated the performance of the activatable EpCAM probe in the context of cancer cell imaging, but we believe the potential applications of fluorogenic probes for antigens are extensive. As the repertoire of targetable antigens expands, fluorogenic probes based on this design could be widely used for diagnostic purposes or for basic biology research that would benefit from wash-free antigen staining.^20,42^ Considering that DARPins for different antigens share a very similar scaffold structure, it might be possible to obtain good probes for other antigens by means of focused, relatively small-scale screening after in silico prediction, though computational requirements might be substantial. In addition, other types of antigen-binding proteins could be employed with the same design principle, as long as the reaction of a thiol and a thiol-reactive fluorophore can be precisely controlled.^43^

Although biologically relevant thiols (e.g., glutathione) are generally present at low concentrations in the extracellular space, and we have demonstrated that our probe is effective in detecting cell–surface antigens, a potential limitation of the developed probes would be that the SiP conjugated with DARPin may undergo undesired intermolecular reactions with thiols in the surrounding environment. Indeed, while the fluorescence of DARPin-SiP conjugates could tolerate up to 0.1% BSA, an irrelevant protein, we found that the fluorescence of DARPin-SiP conjugate could be quenched by increasing the concentration of thiol in the environment, which indicates that the probe is not suitable for use in an intracellular environment where a high concentration of glutathione is present (Figure S15). An approach to solve this issue might be optimizing the reactivity of the fluorophore with thiols. We have previously shown that the reactivity of pyronine/rhodamine derivatives with thiols can be systematically controlled by changing the functional group attached to the 9′ position and the atom at the 10′ position from Si to carbon in the xanthene moiety, as well as changing the substituents of the amino group.^32,44^ Therefore, screening with different fluorophores might be effective to simultaneously achieve high fluorogenicity in response to antigens and high tolerance to thiols in the environment,^32^ and furthermore might enable the development of a range of differently colored fluorogenic probes for antigens.

For further improvement of the probe performance, the effect of introducing Cys around the antigen recognition site of the antigen-binding protein DARPin may need to be considered. As described earlier, the *K*_d_ values of the EGFP probe and the EpCAM probe are larger than those of the original DARPins. This may be because the incorporation of Cys around the loop region itself affects the binding affinity, or because the installed fluorophore affects the binding affinity. While we have demonstrated that the current EpCAM probe is already effective for wash-free cancer imaging, further optimization should be feasible. In this context, we have previously shown that increasing the number of antigen-recognizing loop sequences in a DARPin significantly improves the binding affinity against the target antigen,^45^ so this strategy might be used to improve the probe performance. In addition, quality control of the probe will be a critical factor for practical applications. At present, the probe is purified using affinity-based separation followed by filter-based size exclusion (see Figure S16 for characterization of the developed activatable and always-on probes), and used immediately after preparation to avoid loss of quality, such as time-dependent disulfide bond formation. However, more rigorous purification methods (e.g., liquid chromatography) may be necessary to ensure complete removal of unreacted dye and unmodified DARPin. Furthermore, identifying optimal storage conditions would be important to promote wide usage of the developed probes.

As discussed so far, the direct translation of the target-antigen-binding event to the output of fluorescence enhancement is expected to be useful from both biological and chemical points of view. Furthermore, from another viewpoint, our finding of different rates of fluorescence enhancement upon reaction with NEM and binding to different targets such as GFP and EpCAM is very interesting. Specifically, while the concentration of the NEM (1 mM) used in our setting should be sufficient to immediately trap free thiol considering the reaction rate (typically 10^2^ to 10^4^/M/s), the fluorescence enhancement of our probes upon antigen recognition was faster than when NEM was added. This means that in addition to the Gibbs free energy change (Δ*G*) between the fluorescently quenched Cys-bound form and the fluorescent Cys-dissociated form (which can be estimated by the QM/MM method), there is also a change of the activation energy controlling the kinetics of the reversible reaction between SiP and thiol upon antigen recognition. Although it is practically difficult to control such factors at present, this kind of change in the kinetics of the reversible reactions of the nonfluorescent and fluorescent molecules could in principle be used for controlling the blinking kinetics of self-blinking probes suitable for super-resolution imaging, as we showed in previous studies with SiP or Si-Rhodamine-based probes.^32,46,47^ Further work to pursue this phenomenon more deeply might well prove fruitful.

In summary, we have developed a new chemical design strategy for fluorogenic probes that directly and rapidly detect the binding of their target antigens via a large increase of absorption and fluorescence by utilizing a thiol-reactive fluorophore and a DARPin with a site-specifically installed Cys residue. The EpCAM probe developed in this study could be useful for cancer imaging, and the chemical design strategy developed here is expected to be applicable to a broad range of target antigens.

## Supplementary Material





## Notes

Methods, Figures (S1–S16), Tables (S1–S3), and Methods for organic synthesis (Scheme S1–S8, Chart S1, S2) (PDF)
Data S1–S18 (Predicted structures of the conjugates (S1–S12) and DNA sequences of representative plasmids (S13–S18)) (ZIP)

## Abbreviations

DARPin: designed ankyrin repeat protein
SiP: silicon-pyronine
QY: quantum yield
GFP: green fluorescent protein
EpCAM: epithelial cell adhesion molecule
QM/MM: quantum mechanics/molecular mechanics
