# Supplementary material for: Design of Antigen-Targeting Fluorogenic Probes Utilizing Intramolecular Addition Reaction of Protein-Dye Hybrids
Source: J Am Chem Soc. 2025 Jul 23;147(34):30684–93. doi: 10.1021/jacs.5c04193 (PMC12396215; doi:10.1021/jacs.5c04193)
Supplement: Supplementary file 1 [file ja5c04193_si_001.pdf]

Supporting Information

Design of Antigen-Targeting Fluorogenic Probes Utilizing Intramolecular Addition Reaction of Protein-Dye Hybrids

Mamiko Nakadate[1], Ryosuke Kojima[1]*, Naoki Seike[2], Ryo Tachibana[2], Kyohhei Fujita[1], Reiko Tsuchiya[1], Mako Kamiya[3], Andreas Plückthun[4], Yasuteru Urano[1,2]*

[1] Graduate School of Medicine, The University of Tokyo, Tokyo 113-0033, Japan.
[2] Graduate School of Pharmaceutical Sciences, The University of Tokyo, 113-0033 Tokyo, Japan.
[3] Laboratory for Chemistry and Life Science, Institute of Integrated Research, Institute of Science Tokyo, 226-8501 Kanagawa, Japan.
[4] Department of Biochemistry, University of Zurich, 8057 Zurich, Switzerland.

*kojima@m.u-tokyo.ac.jp
*uranokun@m.u-tokyo.ac.jp

Inventory

Methods……………………………………………………………………………………………………3

Table S1. Plasmids used in this study…………………………………………………………………………9
Table S2. Oligonucleotides used in this study……………………………………………………………15
Table S3. Sequences of proteins and plasmids relevant to this study. ………………………………………18
Figure S1. Categorization of existing OFF/ON type fluorogenic probes, with examples……………………………19
Figure S2. Example of confirmation of sortase-mediated conjugation of 5-Gly-SiP to DARPin 3G86.32 by SiP fluorescence……………………………………………………………………………………………...20
Figure S3. Fold change of SiP fluorescence of the conjugates of 3G86.32 mutants and 5-Gly SiP after the addition of NEM……………………………………………………………………………………………..…..21
Figure S4. Time course of fold change of fluorescence intensity of the 3G86.32-5-Gly SiP conjugates following the addition of GFP or N-ethylmaleimide…………………………………………………………………22
Figure S5. Results of screening of the GFP probes made with the conjugates of 3G86.32 mutants and 6-Gly SiP ……………………………………………………………………………………………………...….23
Figure S6. Absorption spectra related to the activatable GFP probe……………………………………..24
Figure S7. Determination of the dissociation constant of GFP-activatable probe………………………………..24
Figure S8. Fold change of SiP fluorescence of the conjugates of Ec1 mutants and 5-Gly SiP after the addition of NEM…………………………………………………………………………………………………25
Figure S9. Time course of fold change of fluorescence intensity of the Ec1 K152C-5-Gly SiP conjugate following the addition of EpCAM ECD or N-ethylmaleimide.…………………………………………………...26
Figure S10. Determination of the dissociation constant of the activatable EpCAM probe…………………………27
Figure S11. Confirmation of the expression level of EpCAM in the tested cell lines by Western blotting………...27
Figure S12. Additional information related to imaging of HEK293T cells expressing EpCAM-EGFP….………...28
Figure S13. Additional information related to imaging of Capan-1 and Caco-2 cells……………………….……..30
Figure S14. Fluorescence change upon treating the probes with various concentrations of guanidine……….…….31
Figure S15. Susceptibility of the probe to various concentrations of GSH and BSA……………………………….32
Figure S16. Quality check of the purified DARPin and the probes…………………………………………………32

Methods for organic synthesis (Schemes S1-8, Chart S1, S2)…….…………………………………………….33

Supporting references………………………………………………………………………..........………...40

Methods

Reagents and equipment

Reagents for synthesis and bioassay were purchased from Fujifilm Wako Pure Chemicals, Tokyo Kasei Kogyo, Watanabe Chemical, GE Healthcare Japan, Merck, Nippon Gene, New England Biolab (NEB), Toyobo, BIORAD, Genscript, Sigma-Aldrich and Elabscience® and were used without further purification unless otherwise specified. Purification by column chromatography was performed on a YFLC-AI580 (Yamazen). Reverse-phase liquid chromatography was performed on an Isolera (Biotage). The HPLC system for purification and analysis was composed of a reverse-phase column (GL Science), pump (JASCO PU-2080 or PU-2087) and UV detector (JASCO MD-2010 or MD-2018). ^{1}H NMR and ^{13}C NMR spectra were obtained by using an ADVANCE III 400 (Bruker). Chemical shifts (δ) are given in ppm relative to residual solvents for ^{1}H and ^{13}C. High-resolution mass spectra (HRMS) were obtained on a micrOTOFII (Bruker).

Plasmid construction

-General cloning methods

In general, PCR was performed with Q5 High-Fidelity DNA Polymerase (NEB) or KOD-one PCR master mix (Toyobo) in a Veriti 96-well thermal cycler (Thermo Fisher Scientific). DNA digestion was conducted by using restriction enzymes provided by NEB. Agarose gel electrophoresis was performed with a Mupid-exU (TaKaRa). Gel purification of DNA was performed with the Monarch DNA gel extraction kit (NEB). Ligation High ver. 2 (Toyobo) was used for DNA ligation. Gibson assembly was conducted using Gibson assembly Master Mix (NEB) according to the instructions supplied by the provider. DH5α competent cells, Champion^{TM} DH5α high (Cosmo Bio), were used for regular subcloning. Miniprep of the plasmid DNA was performed with the Monarch Plasmid Miniprep Kit (NEB). Sequences of plasmids were confirmed by Sanger sequencing analysis conducted by Azenta Inc.

-Point mutation of DARPin LPETGG Cys mutant (Quick-Change method).

Point mutation was conducted according to Edelheit et al[1] with some modifications, as follows. Firstly, two separate PCR reactions were performed with each of the forward or reverse primers (precisely, they are not polymerase "chain" reactions) using template DNA (~500 ng) encoding the original DARPin sequence and appropriate primers (see Table S1 and Table S2), with 12-18 cycles. The two amplified products were mixed and heated to 95 °C, then the temperature was gradually lowered to room temperature. This solution was subject to *Dpn*I treatment to deplete the original template, and then transformed into *E. coli*. After this process, the normal miniprep procedure was performed.

-Detailed information and construction method for each plasmid.

The method used for construction of each plasmid is described in Tables S1-3.

Expression and purification of DARPin

BL21(DE3) or ECOS^{TM} Competent *E. coli* BL21 (DE3) cells were transformed with a DARPin expression plasmid (kan^{R}). Typically, the generated colonies were added to 3.5-10 mL of Merck's overnight express LB medium containing 50 μg/mL of kanamycin and incubated at 37°C for about 13 - 16 h. After incubation, the culture medium was centrifuged at 6,000 *g* for 1 minute to precipitate a pellet, and the medium was removed. Merck's BugBuster® Master Mix was added (typically 600 μL to the pellet from 10 mL culture) to lyse the pellet. After shaking, the lysate

was centrifuged at 4°C with 16,000 *g* for 20 min. The supernatant including the DARPin was purified using a His Spin Trap (GE Healthcare Japan) according to the manufacturer's instructions. The buffers used for purification were as follows. Binding buffer: 50 mM HEPES (pH 7.4), 500 mM NaCl, 20 mM imidazole. Elution buffer: 50 mM HEPES (pH 7.4), 500 mM NaCl, 500 mM imidazole. The protein solution was desalted using a Merck Amicon Ultra -0.5, PLBC Ultracel-3 membrane, 10 kDa, by centrifugation at 4°C 14,000 *g* for 10 min with repeated loading of 400 µl of 10 mM HEPES buffer (three times). After desalting, the protein solution was stored at -80°C. The concentration of DARPin was measured by BCA protein assay (ThermoFisher). When necessary, protein expression was scaled up, and a His Trap Fast Start Kit (QIAGEN, with gravity flow) was used for purification. The concentration of purified proteins was measured with Pierce™ BCA Protein Assay Kits (Thermo Fisher Scientific).

Preparation of soluble GFP.

An expression plasmid for His-tagged GFP (pZW16) was transformed into BL21 (DE3) cells (selected with ampicillin). GFP was purified using a similar method to that described for DARPin: Overnight express® LB medium: 50 ml scale, lysis: 3 mL of BugBuster® Master Mix, purification on a His Trap FF (Cytiva) with a manual syringe, followed by desalting with a Merck Amicon Ultra -0.5, PLBC Ultracel-3 membrane, 10 kDa. The resulting GFP solution in 10 mM HEPES (pH 7.4) was stored at -80 °C. The concentration of purified proteins was determined from the absorbance of GFP using the extinction coefficient of 55,900 M^{-1} cm^{-1} at λ_{max}. The concentration determined in this way was confirmed to be similar to that determined with Pierce™ BCA Protein Assay Kits (Thermo Fisher Scientific).

Preparation of sortase A.

An expression plasmid encoding GST-tagged sortase A pentamutant (pRK307) was transformed into BL21 (DE3) (selected with ampicillin). Sortase was expressed in a similar manner to DARPin. After the cell pellet had been lysed with BugBuster®MasterMix (containing 0.1% benzonase nuclease (Merck, 70746) as an additive), the lysate was centrifuged at 16,000 G for 20 min at 4 °C. The supernatant including GST-tagged sortase A was purified on GSTrap FF columns (GE Healthcare Japan) with a manual syringe, using PBS (-) as the equilibration and wash buffer. The GST tag of sortase A was cleaved with 160 units of PreScission protease (Cytiva 27084301) dissolved in PreScission cleavage buffer (50 mM Tris (pH 7.4), 150 mM NaCl, 1 mM EDTA, 1 mM DTT). Sortase A was eluted with PreScission cleavage buffer. Finally, the buffer was exchanged to 10 mM HEPES (pH 7.4) by using a PD 25-MiniTrap. The resulting protein solution was stored at -80°C. The concentration of purified proteins was measured with Pierce™ BCA Protein Assay Kits (Thermo Fisher Scientific).

Conjugate preparation

DARPins were labelled with a SiP derivative bearing poly-glycine by sortase A. A 10x sortase reaction buffer consisting of Tris 500 mM, NaCl 1.5 M and $CaCl_2$ 100 mM, adjusted to pH 7.5 with 1 N HCl, was used after 10-fold dilution with water. DARPin (10-50 µM (1 eq.)), 5 (or 6)-Gly SiP dye (20-100 µM (2 eq.), and sortase A (final conc. 1~2 µM) were mixed in the 1x sortase buffer. The concentration of the dye was calculated from the absorbance in PBS (pH 7.4), assuming an extinction co-efficient of 100,000 M^{-1} cm^{-1} at the λ_{max}. The reaction mixture was incubated

for about 13~16 h at 37°C with rotation, then purified by using a His Spin Trap according to the manufacturer's instructions (the wash buffer and elution buffer are described in the section on protein expression and purification). Finally, the buffer was changed to 10 mM HEPES (pH 7.4) by using a Merck Amicon Ultra-0.5 PLBC Ultracel-3 membrane, NMWCO 10 kDa (we typically performed three washes consisting of the following steps: add 450 μl of 10 mM HEPES, and centrifuge at 14,000 G for 30 min). After preparation, the conjugate was stored at 4°C and used as soon as possible (typically on the same day or, at the latest, within two days).

SDS-PAGE

Purification of the protein and conjugation of the dye to DARPin were monitored by SDS-PAGE. 10x SDS PAGE buffer (Tris 30.3 g, glycine 144.0 g, SDS 10.0 g dissolved in Milli Q, adjusted to pH 8.3, and then made up to 1 L with Milli Q) was used after 10-fold dilution. To prepare a loading sample, 5 μL of loading buffer (4× Laemmli sample buffer: 2-mercaptoethanol = 9:1) was added to 15 μl of the sample, and the mixture was heated at 95 °C for about 5 min (in the case of reduced and boiled samples). The samples were loaded onto MiniProtian TGX gel (BIORAD). Electrophoresis was conducted on a PowerPacHC (BIORAD). Fluorescence was analyzed with a LAS4000 (GE Healthcare), FUSION FX (Vilber Bio Imaging) or an iBright FL1500 Imaging System (Thermofisher). For protein visualization, the gel was stained with Coomassie Brilliant Blue G-250 (Tokyo Kasei), and monitored with the same instruments used for observing in-gel fluorescence.

Screening of activatable probes

An Envision plate 2103 multilabel reader was used to monitor fluorescence. Excitation filter: 620 nm (±5 nm), emission filter: 685 nm (±17.5 nm). 20 μL of 10 mM HEPES buffer (pH 7.4, 0.5% CHAPS) containing 0.5 μM conjugate (without DTT pretreatment) (or blank) was dispensed into a 384-well plate (Corning, 4514), and fluorescence measurement was started. After 15-30 min, 10 mM HEPES buffer (pH 7.4) containing recombinant GFP (final conc. 1 μM), EpCAM ECD (final conc. 1.5 μM recombinant human EpCAM/TROP-1 protein (Fc Tag) Elabscience, PKSH032383), or *N*-ethylmaleimide (final conc. 1 mM) was added. When antigen/NEM solution was added, 10x solution was used so that the change of volume was small. Fluorescence intensity was monitored every 5 min. The time point of 0 min indicates the point just before the addition of the antigen or NEM, and the time point of 1 min indicates the point just after the addition of the antigen or NEM.

Measurement of photophysical properties of GFP probes

Absorption spectra were obtained on a UV-2450 (Shimadzu). Fluorescence spectra were obtained with an F-7000 instrument (Hitachi). Quantum yield of fluorescence was measured with a Quantaurus-QY (Hamamatsu Photonics). For measuring absorption and fluorescence spectra, a micro quartz cuvette (Hellma105-250-15-40) was used. For measuring QY of fluorescence, a specialized quartz cuvette matching the instrument was used (Hamamatsu Photonics, A10095-02).

The absorbance and fluorescence spectra of 0.5 μM conjugate in 10 mM HEPES buffer (pH 7.4, containing 0.5 % CHAPS as an additive) were measured. Then, self-purified GFP was added to the conjugate solution at a final concentration of 1 μM. Absorbance and fluorescence spectra were obtained at 1 hr after adding GFP.

DTT pretreatment of the conjugate

10 mM DTT was added to the purified DARPin-SiP conjugate and the mixture was incubated at 37℃ for 1 hr. Then, DTT was completely removed by changing the buffer to 10 mM HEPES (pH 7.4) with a Merck Amicon Ultra-0.5 PLBC Ultracel-3 membrane, NMWCO 10 kDa. The DTT-pretreated conjugates were immediately used for the following experiments.

Cell culture

All cells were maintained at 37°C in a humidified atmosphere containing 5% CO_2. Culture media were as follows. HEK293T cells (Riken BRC Cell Bank (Tsukuba, Japan), RCB2202): Dulbecco's modified Eagle's medium (DMEM, high glucose with L-glutamate, FUJIFILM Wako) with 10% (v/v) fetal bovine serum (FBS, Biosera) and 1 % (v/v) penicillin/streptomycin solution (PS, FUJIFILM Wako). Caco-2 cells (originally from ATCC (VA, USA, HTB-37), gift from Dr. Yusuke Yoshioka, Tokyo Medical University): MEM (Gibco) with 10% FBS, 1x non-essential amino acids (NEAA, FUJIFILM Wako) and 1% PS. Capan-1 cells (originally from ATCC (VA, USA, HTB-79), gift from Dr. Yusuke Yoshioka, Tokyo Medical University): IMDM (with L-glutamate, HEPES, and sodium pyruvate, FUJIFILM Wako) with 20% FBS and 1% PS. Cells were split when they reach approximately 80 % confluency. The split ratio was determined taking account of the doubling time of the cells, so that splitting was done approximately every 3 days.

Establishment of stable cell lines

In this study, HEK293T cells stably expressing EpCAM-EGFP or cytosolic EGFP were constructed. For this purpose, the Sleeping Beauty transposase system was used. HEK293T cells were plated at 2.5 x 10^5 cells/mL onto wells of 12-well plates (in 1 mL of medium) and cultured for 24 hours before transfection. DNA mix containing 50 ng of transposase-encoding plasmid (addgene #34879, pCMV(CAT)T7-SB100) and 950 ng of transposon plasmid (pMN227 (see Tables S1, S3) for expressing EpCAM-EGFP, or pRK393 (see Table S1) for expressing cytosolic EGFP) in 100 µl OptiMEM was prepared, and then 4 µl of Polyethyleneimine "Max" (PEI, Polyscience #24765, 1 mg/mL in dH_2O) was added. The mixture was briefly vortexed and then incubated at room temperature for 15 min. In parallel, the cell culture medium was renewed before transfection. Then, the DNA/PEI mixture was added dropwise to the culture. After a sufficient cultivation period (typically 16 h), the cell culture medium was renewed. At 48 hrs after transfection, appropriate antibiotics (0.5 µg/mL puromycin for the expression of EpCAM-EGFP by pMN227, 300 µg/mL hygromycin B for the expression of cytosolic GFP by pRK393) were added to the cell culture medium to start selection. Sufficiently expanded cells were confirmed to express the desired protein by measuring their green fluorescence.

Western blotting

Whole-cell lysates were prepared using RIPA buffer (cat. #182-02451, Wako FujiFilm). The lysates were denatured under non-reducing conditions. Five micrograms of each protein sample was separated by SDS-PAGE and transferred to PVDF membranes using the iBlot 3 Western Blot Transfer System (cat. #34002, Invitrogen, Thermo Fisher

Scientific). The membranes were blocked with Blocking One (cat. #03953, Nacalai Tesque) for 30 minutes at room temperature (RT), followed by incubation with an anti-EpCAM primary antibody (1:1000, cat. #2929, Cell Signaling Technology) at 4°C overnight. After washing, the membranes were incubated for 1 hour at RT with an HRP-conjugated secondary antibody (1:2000, cat. #7076, Cell Signaling Technology). The signal was detected using ECL substrate (cat. #RPN2232V1, RPN2232V2, Cytiva) and the iBright FL1500 Imaging System (Thermo Fisher Scientific). The membranes were then treated with stripping buffer (cat. #T7135A, Takara) and reprobed for endogenous actin using an anti-β-actin antibody (1:2000, cat. #20536-1-AP, ProteinTech) and a secondary antibody (1:2000, cat. #7074, Cell Signaling Technology).

Live cell imaging

HEK293T cells expressing EpCAM-EGFP, Capan-1 cells and Caco-2 cells were seeded in ibidi 8-well chambers, and cultured with corresponding culture media for at least 24 hours before imaging. For the mixed culture of Capan-1 cells or Caco-2 cells with HEK293T cells expressing cytosolic GFP, the media for Capan-1 cells or Caco-2 cells were used, respectively. For imaging, the medium was removed from the 8-well chambers, and 250 ml of DPBS (-) (Gibco), HBSS(-, no phenol red) (Gibco), OptiMEM (Gibco) or DMEM with 10% FBS, containing 500 nM probe (0.01 % CHAPS as an additive) was added to the cells in each well. The cells were imaged within 30 min by a Leica TCS SP5. Conditions for the fluorescence observation are given in the figure legends. Note that the look-up tables cannot be directly compared between subfigures, due to the difference of microscope settings (e.g., laser intensity).

Structure calculation

-MD sampling

The structure of a DARPin and GFP pair (PDBID: 6MWQ[2], Chain A/G) was extracted. For the DARPin part, the N- and C-terminal amino acid structures were added or cut so that the sequence matched the experimentally produced DARPin. Here, the structure of SiP was also appended. The amino acid residues were changed manually to account for the mutation (151-153C) from the original structure. For each of 6 kinds of structure (3 mutations, GFP(+/-)), umbrella sampling was performed with GROMACS 2023.1.[3] All titratable amino acids were assigned their canonical state at physiological pH. Parameters generated by Automated Topology Builder[4] were set for SiP and its adjoining glycine. Parameters from the GROMOS 54A7 parameter set were applied to all other amino acids in the simulated system. The detailed sampling settings were based on literature methodology[5]. The distance between the sulfur atom of the cysteine residue and the carbon atom of the SiP under nucleophilic attack was used as a constraint, and the structure was sampled so that the SiP was closer to the cysteine. From this trajectory, structures with the S-C distance of 36 Å (as the ON state) and 3 Å (as the OFF state) were extracted. Then, using these as the initial structures, the NPT ensembles were obtained with the S-C distance fixed, followed by 4 ns of sampling. This trajectory was analyzed in clusters and the representative major structure was extracted.

-QM/MM calculation

Ions and solvent molecules at distances greater than 15 Å from both SiP and the cysteine were removed from the MD structure, and for the OFF state, the positions of SiP and cysteine residues were fine-tuned to form a chemical bond between them. Protons at pH 7.4 were assigned again to each amino acid using the PROPKA server.[6] The structures

were optimized by the 2-layer ONIOM method with Gaussian16.[7] The calculations were performed at the level of ONIOM(B3LYP/6-31G(d,p):UFF). The high layer included the SiP moiety (to the carbon unit adjacent to the amino groups), the side chain of the cysteine and three water molecules which directly interact with SiP via hydrogen bonds. For the low layer, the atomic charge of the SiP moiety was calculated at the same level as for the high layer. In the structural optimization, only the atoms within 8 Å of the high layer atoms, except for the oxygen atoms of the solvent molecules in the lower layers, were allowed to relax. The energy of the optimized structures was further calculated at the level of ONIOM(APFD/ 6-311+g(2d,p):UFF) including electronic embedding. The code used for predicting the developed protein-dye hybrids is available on GitHub at https://github.com/TachibanaRyo-moroba/DARPin-SiP.

Determining the K_d values of the probes for the target antigen.

The K_d values of the fluorogenic probes were calculated from the change of fluorescence upon antigen addition, which was measured with an Envision multi-well plate reader (Perkin Elmer). Various concentrations of target antigen were prepared and added to 20 µL of 50 nM conjugate (generally, stock solution of the target antigen was more than 10 times concentrated as compared with the desired final concentration to avoid drastic changes of the volume). Fluorescence intensity was measured before the addition of the antigen and when the fluorescence intensity had reached a plateau after antigen addition. Kaleida graph was used to create the titration curve, and the K_d of the conjugate for the antigen was calculated by applying the following equation[8].

$$Y = \frac{(F_{max} - F_0)\left((C + X + K_d) - \sqrt{(C + X + K_d)^2 - 4 \times C \times X}\right)}{2 \times C}$$

F_{max}; Maximum fluorescence intensity at a saturated concentration of antigen. F_0; Fluorescence intensity without antigen. C; Total concentration of the conjugate. X; Concentration of the antigen. Y: Fluorescence increase measured at each concentration of the antigen. K_d; The binding affinity to be estimated.

Note regarding the drawing of the protein cartoons in the figures

For GFP, the cartoon was written based on PDB 2Y0G. For the anti-GFP DARPin, the structure was written based on PDB 6MWQ (taking out only anti-GFP DARPin). For anti-EpCAM DARPin, the cartoon was written based on the structure predicted using AlphaFold2 (ColabFold v1.5.5).

Supporting Tables

Plasmid	Description and Cloning Strategy	Reference/Source
**Sortase A pentamutant (eSrtA) in pET29**	Constitutive expression vector of $(His)_6$-tagged sortase A. Applicable plasmid was bought from addgene (addgene number: 75144[9]).	Addgene
**pGEX-2T**	An empty plasmid having a GST tag.	GE Healthcare Japan
**pSBbi-Pur**	A plasmid encoding Sleeping Beauty transposon with bi-directional promoter (addgene number: 60523[10]).	Addgene
**pCMV(CAT)T7-SB100**	A plasmid encoding Sleeping Beauty transposase vector (addgene number: 34879[11]).	Addgene
**pRK202**	A template plasmid encoding wild-type DARPin Ec1 used in this study. This plasmid was constructed by using a DARPin Ec1-encoding plasmid[12] provided by Andreas Plückthun.	Unpublished
**pRK307**	Expression vector of GST tag-sortase A with PreScission protease site between GST tag and sortase A. The sortase A region was PCR amplified from sortase A pentamutant (eSrtA) in pET29 (addgene 75144) by oMN69 (encoding PreScission protease site) and oMN70. The amplified DNA fragment was digested with *EcoR*I-HF/*BamH*I-HF and inserted into corresponding site of pGEX-2T.	This work
**pRK187**	A plasmid for constitutive expression of EpCAM and ZsGreen.	Kojima et al[13].
**pRK393**	Expression vector of cytosolic EGFP (together with firefly luciferase luc2) with Sleeping Beauty transposon. Luc2 encoding sequence was inserted into pSBbi-GH (addgene #60514) digested with SfiI. Full sequence is available at https://benchling.com/s/seq-xCTh2EK9dLICBuXcI1Hq?m=slm-YZQxWbmp0p6nWJLqmZ1v and Data S13.	This work
**pZW16**	Expression vector of $(His)_6$-tagged GFP. A fragment of EGFP was amplified from pEGFP-C1 by oZW25 and oZW26. The amplicon was digested with *Xba*I and *EcoR*I and inserted into the corresponding site of pZW13 (his-TEV-GGGS-Td2F2 expression plasmid, unpublished. This plasmid has the same backbone as pGEX-2T).	This work
**pMN1**	Expression vector of $(His)_6$-tagged DARPin G3 in pCold I. DARPin G3 was amplified from pRK176 (unpublished, encoding DARPin G3[14] sequence provided by Andreas Plückthun) by oMN1 and oMN2. The amplicon was digested with *Ase*I/*Xba*I and inserted into corresponding site of pCold I (Takara Bio). This plasmid is an intermediate plasmid for subcloning.	This work
**pMN8**	Expression vector of $(His)_6$-tagged DARPin G3 LPETGG. $(His)_6$-tagged DARPin G3 LPETGG was amplified from pMN1 by using oMN11 and oMN14. The amplified DNA fragment was digested with *Nde*I/*Xho*I and inserted into corresponding site of sortase A pentamutant (eSrtA) in pET29 (addgene #75144) (replacing sortase). This plasmid is an intermediate plasmid for subcloning.	This work

**pMN37**	Expression vector of $(His)_6$-tagged DARPin Ec1 LPETGG. $(His)_6$-tagged DARPin Ec1 LPETGG was digested from pRK202 by using *BamH*I-HF and *Hind*III-HF. The digested DNA fragment was inserted into the corresponding site of pMN8. **The amino acid sequence of the protein expressed by this plasmid can be found in Table S3.** Full DNA sequence of pMN37 is available at: https://benchling.com/s/seq-vhOBAroGyNgELWVAcIpM?m=slm-qcm3nfExaCFoneCwA2GU and Data S14.	This work
**pMN39**	Expression vector of $(His)_6$-tagged DARPin 3G86.32 LPETGG. $(His)_6$-tagged DARPin 3G86.32 LPETGG was digested from DNA fragment including 3G86.32[15] (provided by Andreas Plückthun) by using *BamH*I-HF and *Hind*III-HF. The digested DNA fragment was inserted into the corresponding site of pMN8.	This work
**pMN43**	Expression vector of $(His)_6$-tagged DARPin 3G86.32 C76N LPETGG. C76 was converted to N considering that many DARPins bear N at this position, and this position was expected to be structurally irrelevant to the antigen binding. The insert was constructed from pMN39 by the Quick-Change method using oMN87 and oMN88. Hereafter, C76N is always installed (pMN45-64), and the description of C76N is omitted to avoid confusion. **The amino acid sequence of the protein expressed by this plasmid can be found in Table S3.** Full DNA sequence of pMN43 is available at: https://benchling.com/s/seq-WRC39716rc0bxjLTq7FY?m=slm-jW4zfwxW9WnzYyIYUWLT and Data S15.	This work
**pMN45**	Expression vector of $(His)_6$-tagged DARPin 3G86.32 LPETGG N147C. The insert was constructed from pMN43 by the Quick-Change method using oMN91 and oMN92.	This work
**pMN46**	Expression vector of $(His)_6$-tagged DARPin 3G86.32 LPETGG A148C. The insert was constructed from pMN43 by the Quick-Change method using oMN93 and oMN94.	This work
**pMN47**	Expression vector of $(His)_6$-tagged DARPin 3G86.32 LPETGG Q149C. The insert was constructed from pMN43 by the Quick-Change method using oMN95 and oMN96.	This work
**pMN48**	Expression vector of $(His)_6$-tagged DARPin 3G86.32 LPETGG D150C. The insert was constructed from pMN43 by the Quick-Change method using oMN97 and oMN98.	This work
**pMN49**	Expression vector of $(His)_6$-tagged DARPin 3G86.32 LPETGG K151C. The insert was constructed from pMN43 by the Quick-Change method using oMN99 and oMN100.	This work
**pMN50**	Expression vector of $(His)_6$-tagged DARPin 3G86.32 LPETGG F152C. The insert was constructed from pMN43 by the Quick-Change method using oMN101 and oMN102. This plasmid is designed for constructing the activatable GFP probe. Full DNA sequence of pMN50 is available at: https://benchling.com/s/seq-v4aoCjOwl3siXITbZRs0?m=slm-SjflV9SIHc858y2vlNoO and Data S16.	This work

**pMN51**	Expression vector of $(His)_6$-tagged DARPin 3G86.32 LPETGG G153C. The insert was constructed from pMN43 by the Quick-Change method using oMN103 and oMN104.	This work
**pMN52**	Expression vector of $(His)_6$-tagged DARPin 3G86.32 LPETGG K154C. The insert was constructed from pMN43 by the Quick-Change method using oMN105 and oMN106.	This work
**pMN53**	Expression vector of $(His)_6$-tagged DARPin 3G86.32 LPETGG T155C. The insert was constructed from pMN43 by the Quick-Change method using oMN107 and oMN108.	This work
**pMN54**	Expression vector of $(His)_6$-tagged DARPin 3G86.32 LPETGG A156C. The insert was constructed from pMN43 by the Quick-Change method using oMN109 and oMN110.	This work
**pMN55**	Expression vector of $(His)_6$-tagged DARPin 3G86.32 LPETGG F157C. The insert was constructed from pMN43 by the Quick-Change method using oMN111 and oMN112.	This work
**pMN56**	Expression vector of $(His)_6$-tagged DARPin 3G86.32 LPETGG D158C. The insert was constructed from pMN43 by the Quick-Change method using oMN113 and oMN114.	This work
**pMN57**	Expression vector of $(His)_6$-tagged DARPin 3G86.32 LPETGG I159C. The insert was constructed from pMN43 by the Quick-Change method using oMN115 and oMN116.	This work
**pMN58**	Expression vector of $(His)_6$-tagged DARPin 3G86.32 LPETGG S160C. The insert was constructed from pMN43 by the Quick-Change method using oMN117 and oMN118.	This work
**pMN59**	Expression vector of $(His)_6$-tagged DARPin 3G86.32 LPETGG I161C. The insert was constructed from pMN43 by the Quick-Change method using oMN119 and oMN120.	This work
**pMN60**	Expression vector of $(His)_6$-tagged DARPin 3G86.32 LPETGG D162C. The insert was constructed from pMN43 by the Quick-Change method using oMN121 and oMN122.	This work
**pMN61**	Expression vector of $(His)_6$-tagged DARPin 3G86.32 LPETGG N163C. The insert was constructed from pMN43 by the Quick-Change method using oMN123 and oMN124.	This work

**pMN62**	Expression vector of $(His)_6$-tagged DARPin 3G86.32 LPETGG G164C. The insert was constructed from pMN43 by the Quick-Change method using oMN125 and oMN126.	This work
**pMN63**	Expression vector of $(His)_6$-tagged DARPin 3G86.32 LPETGG N165C. The insert was constructed from pMN43 by the Quick-Change method using oMN127 and oMN128.	This work
**pMN64**	Expression vector of $(His)_6$-tagged DARPin 3G86.32 LPETGG E166C. The insert was constructed from pMN43 by the Quick-Change method using oMN129 and oMN130.	This work
**pMN147**	A Sleeping Beauty transposon plasmid for constitutive expression of HER2-EGFP fusion protein in mammalian cells. Firstly, pMN33 (encoding HER2-EGFP in pcDNA3.1) was constructed by replacing iRFP720 of pRK21 (encoding HER2-iRFP720 in pRK21[13]) by normal restriction cloning. Then, the fragment coding HER2-EGFP was digested out by *Nco*I-HF/*Xba*I and was inserted into the corresponding site of pSBbi-pur (addgene 60523). This plasmid is an intermediate plasmid for subcloning.	This work
**pMN203**	Expression vector of $(His)_6$-tagged DARPin Ec1 LPETGG V144C. The insert was constructed from pMN37 by the Quick-Change method using oMN324 and oMN325.	This work
**pMN204**	Expression vector of (His)6-tagged DARPin Ec1 LPETGG N145C. The insert was constructed from pMN37 by the Quick-Change method using oMN326 and oMN327.	This work
**pMN205**	Expression vector of (His)6-tagged DARPin Ec1 LPETGG A146C. The insert was constructed from pMN37 by the Quick-Change method using oMN328 and oMN329.	This work
**pMN206**	Expression vector of (His)6-tagged DARPin Ec1 LPETGG Q147C. The insert was constructed from pMN37 by the Quick-Change method using oMN330 and oMN331.	This work
**pMN207**	Expression vector of (His)6-tagged DARPin Ec1 LPETGG D148C. The insert was constructed from pMN37 by the Quick-Change method using oMN332 and oMN333.	This work
**pMN208**	Expression vector of (His)6-tagged DARPin Ec1 LPETGG R149C. The insert was constructed from pMN37 by the Quick-Change method using oMN334 and oMN335.	This work

**pMN209**	Expression vector of (His)6-tagged DARPin Ec1 LPETGG S150C. The insert was constructed from pMN37 by the Quick-Change method using oMN336 and oMN337.	This work
**pMN210**	Expression vector of (His)6-tagged DARPin Ec1 LPETGG G151C. The insert was constructed from pMN37 by the Quick-Change method using oMN338 and oMN339.	This work
**pMN211**	Expression vector of (His)6-tagged DARPin Ec1 LPETGG K152C. The insert was constructed from pMN37 by the Quick-Change method using oMN340 and oMN341. This plasmid is designed for constructing the activatable EpCAM probe. Full DNA sequence is available at: https://benchling.com/s/seq-cPtqgZhnMLZicdGGVYec?m=slm-DttCVG2n1jQz14w1bAoP and Data S17.	This work
**pMN212**	Expression vector of (His)6-tagged DARPin Ec1 LPETGG T153C. The insert was constructed from pMN37 by the Quick-Change method using oMN342 and oMN343.	This work
**pMN213**	Expression vector of (His)6-tagged DARPin Ec1 LPETGG P154C. The insert was constructed from pMN37 by the Quick-Change method using oMN344 and oMN345.	This work
**pMN214**	Expression vector of (His)6-tagged DARPin Ec1 LPETGG F155C. The insert was constructed from pMN37 by the Quick-Change method using oMN346 and oMN347.	This work
**pMN215**	Expression vector of (His)6-tagged DARPin Ec1 LPETGG D156C. The insert was constructed from pMN37 by the Quick-Change method using oMN348 and oMN349.	This work
**pMN216**	Expression vector of (His)6-tagged DARPin Ec1 LPETGG L157C. The insert was constructed from pMN37 by the Quick-Change method using oMN350 and oMN351.	This work
**pMN217**	Expression vector of (His)6-tagged DARPin Ec1 LPETGG A158C. The insert was constructed from pMN37 by the Quick-Change method using oMN352 and oMN353.	This work
**pMN218**	Expression vector of (His)6-tagged DARPin Ec1 LPETGG I159C. The insert was constructed from pMN37 by the Quick-Change method using oMN354 and oMN355.	This work
**pMN219**	Expression vector of (His)6-tagged DARPin Ec1 LPETGG D160C. The insert was constructed from pMN37 by the Quick-Change method using oMN356 and oMN357.	This work

**pMN220**	Expression vector of (His)6-tagged DARPin Ec1 LPETGG N161C. The insert was constructed from pMN37 by the Quick-Change method using oMN358 and oMN359.	This work
**pMN221**	Expression vector of (His)6-tagged DARPin Ec1 LPETGG G162C. The insert was constructed from pMN37 by the Quick-Change method using oMN360 and oMN361.	This work
**pMN222**	Expression vector of (His)6-tagged DARPin Ec1 LPETGG N163C. The insert was constructed from pMN37 by the Quick-Change method using oMN362 and oMN363.	This work
**pMN223**	Expression vector of (His)6-tagged DARPin Ec1 LPETGG E164C. The insert was constructed from pMN37 by the Quick-Change method using oMN364 and oMN365.	This work
**pMN227**	Expression vector of EpCAM-EGFP fusion protein with Sleeping Beauty transposon. The EpCAM-encoding DNA fragment was amplified from pRK187 and inserted into pMN147 (digested with *Nco*I-HF and *Not*I-HF) by Gibson assembly. **The amino acid sequence of the protein expressed by this plasmid can be found in Table S3.** Full DNA sequence of pMN37 is available at: https://benchling.com/s/seq-vbU16uKIPO5jg7rUQAuf?m=slm-PAvQexXAisNN4yIU0FKI and Data S18.	This work

Table S1. List of plasmids used in this study.

Oligo number	Sequence (5'→3')
**oMN1**	GACTATTAATGGATCCGACCTGGGTAAGAAACTACTGGAAGCTG
**oMN2**	ATGCTCTAGACTAATTAAGCTTTTGCAGGATTTCAGCCAGGTCCTCG
**oMN11**	ATTACTCGAGCTAACCGCCTGTTTCAGGAAGATTAAGCTTTTGCAGGATTTCAGCCAGGTCCTCGTTAC
**oMN14**	TATACATATGAATCACAAAGTGCATCATCATCATCATCATATCGAAGGTAGGCATAATGGATCCGACCTG
**oMN69**	ATTAGGATCCGATCTGGAAGTTCTGTTCCAGGGGCCCATGCAAGCTAAACCTCAAATTCCGAAAGATAAATC
**oMN70**	ATTAGAATTCATTTGACTTCTGTAGCTACAAAGATTTTACGTGTTTCC
**oMN87**	GTTGAAGTTCTGCTGAAGAACGGTGCTGACGTTAACGCTGCTGAC
**oMN88**	GTCAGCAGCGTTAACGTCAGCACCGTTCTTCAGCAGAACTTCAAC
**oMN91**	CACGGTGCTGACGTTTGCGCTCAGGACAAATTC
**oMN92**	GAATTTGTCCTGAGCGCAAACGTCAGCACCGTG
**oMN93**	GGTGCTGACGTTAACTGCCAGGACAAATTCGGT
**oMN94**	ACCGAATTTGTCCTGGCAGTTAACGTCAGCACC
**oMN95**	GGTGCTGACGTTAACGCTTGCGACAAATTCGGTAAGACC
**oMN96**	GGTCTTACCGAATTTGTCGCAAGCGTTAACGTCAGCACC
**oMN97**	CTGACGTTAACGCTCAGTGCAAATTCGGTAAGACCGC
**oMN98**	GCGGTCTTACCGAATTTGCACTGAGCGTTAACGTCAG
**oMN99**	GACGTTAACGCTCAGGACTGCTTCGGTAAGACCGCTTTC
**oMN100**	GAAAGCGGTCTTACCGAAGCAGTCCTGAGCGTTAACGTC
**oMN101**	GTTAACGCTCAGGACAAATGCGGTAAGACCGCTTTCGAC
**oMN102**	GTCGAAAGCGGTCTTACCGCATTTGTCCTGAGCGTTAAC
**oMN103**	AACGCTCAGGACAAATTCTGCAAGACCGCTTTCGACATC
**oMN104**	GATGTCGAAAGCGGTCTTGCAGAATTTGTCCTGAGCGTT
**oMN105**	GCTCAGGACAAATTCGGTTGCACCGCTTTCGACATCTCC
**oMN106**	GGAGATGTCGAAAGCGGTGCAACCGAATTTGTCCTGAGC
**oMN107**	TCAGGACAAATTCGGTAAGTGCGCTTTCGACATCTCCATCG
**oMN108**	CGATGGAGATGTCGAAAGCGCACTTACCGAATTTGTCCTGA
**oMN109**	GGACAAATTCGGTAAGACCTGCTTCGACATCTCCATCGACA
**oMN110**	TGTCGATGGAGATGTCGAAGCAGGTCTTACCGAATTTGTCC
**oMN111**	CAAATTCGGTAAGACCGCTTGCGACATCTCCATCGACAATG
**oMN112**	CATTGTCGATGGAGATGTCGCAAGCGGTCTTACCGAATTTG
**oMN113**	TTCGGTAAGACCGCTTTCTGCATCTCCATCGACAATGGT

**oMN114**	ACCATTGTCGATGGAGATGCAGAAAGCGGTCTTACCGAA
**oMN115**	GGTAAGACCGCTTTCGACTGCTCCATCGACAATGGTAAC
**oMN116**	GTTACCATTGTCGATGGAGCAGTCGAAAGCGGTCTTACC
**oMN117**	AAGACCGCTTTCGACATCTGCATCGACAATGGTAACGAG
**oMN118**	CTCGTTACCATTGTCGATGCAGATGTCGAAAGCGGTCTT
**oMN119**	CCGCTTTCGACATCTCCTGCGACAATGGTAACGAGGA
**oMN120**	TCCTCGTTACCATTGTCGCAGGAGATGTCGAAAGCGG
**oMN121**	CGCTTTCGACATCTCCATCTGCAATGGTAACGAGGACCTGG
**oMN122**	CCAGGTCCTCGTTACCATTGCAGATGGAGATGTCGAAAGCG
**oMN123**	CGACATCTCCATCGACTGCGGTAACGAGGACCTGG
**oMN124**	CCAGGTCCTCGTTACCGCAGTCGATGGAGATGTCG
**oMN125**	GACATCTCCATCGACAATTGCAACGAGGACCTGGCTGAA
**oMN126**	TTCAGCCAGGTCCTCGTTGCAATTGTCGATGGAGATGTC
**oMN127**	TCCATCGACAATGGTTGCGAGGACCTGGCTGAA
**oMN128**	TTCAGCCAGGTCCTCGCAACCATTGTCGATGGA
**oMN129**	TCCATCGACAATGGTAACTGCGACCTGGCTGAAATCCTG
**oMN130**	CAGGATTTCAGCCAGGTCGCAGTTACCATTGTCGATGGA
**oMN324**	CTGAAGAATGTTGCTGACTGCAACGCTCAGGACAGATCC
**oMN325**	GGATCTGTCCTGAGCGTTGCAGTCAGCAACATTCTTCAG
**oMN326**	AAGAATGTTGCTGACGTTTGCGCTCAGGACAGATCCGGT
**oMN327**	ACCGGATCTGTCCTGAGCGCAAACGTCAGCAACATTCTT
**oMN328**	AAGAATGTTGCTGACGTTAACTGCCAGGACAGATCCGGTAAGACC
**oMN329**	GGTCTTACCGGATCTGTCCTGGCAGTTAACGTCAGCAACATTCTT
**oMN330**	GTTGCTGACGTTAACGCTTGCGACAGATCCGGTAAGACC
**oMN331**	GGTCTTACCGGATCTGTCGCAAGCGTTAACGTCAGCAAC
**oMN332**	CTGACGTTAACGCTCAGTGCAGATCCGGTAAGACCCC
**oMN333**	GGGGTCTTACCGGATCTGCACTGAGCGTTAACGTCAG
**oMN334**	GTTAACGCTCAGGACTGCTCCGGTAAGACCCCG
**oMN335**	CGGGGTCTTACCGGAGCAGTCCTGAGCGTTAAC
**oMN336**	GTTAACGCTCAGGACAGATGCGGTAAGACCCCGTTCGAC
**oMN337**	GTCGAACGGGGTCTTACCGCATCTGTCCTGAGCGTTAAC
**oMN338**	CGCTCAGGACAGATCCTGCAAGACCCCGTTCGACT

**oMN339**	AGTCGAACGGGGTCTTGCAGGATCTGTCCTGAGCG
**oMN340**	CAGGACAGATCCGGTTGCACCCCGTTCGACTTAGCG
**oMN341**	CGCTAAGTCGAACGGGGTGCAACCGGATCTGTCCTG
**oMN342**	CAGGACAGATCCGGTAAGTGCCCGTTCGACTTAGCGATC
**oMN343**	GATCGCTAAGTCGAACGGGCACTTACCGGATCTGTCCTG
**oMN344**	CAGGACAGATCCGGTAAGACCTGCTTCGACTTAGCGATCGACAAC
**oMN345**	GTTGTCGATCGCTAAGTCGAAGCAGGTCTTACCGGATCTGTCCTG
**oMN346**	AGATCCGGTAAGACCCCGTGCGACTTAGCGATCGACAAC
**oMN347**	GTTGTCGATCGCTAAGTCGCACGGGGTCTTACCGGATCT
**oMN348**	CCGGTAAGACCCCGTTCTGCTTAGCGATCGACAACGG
**oMN349**	CCGTTGTCGATCGCTAAGCAGAACGGGGTCTTACCGG
**oMN350**	GACCCCGTTCGACTGCGCGATCGACAACG
**oMN351**	CGTTGTCGATCGCGCAGTCGAACGGGGTC
**oMN352**	AAGACCCCGTTCGACTTATGCATCGACAACGGTAACGAG
**oMN353**	CTCGTTACCGTTGTCGATGCATAAGTCGAACGGGGTCTT
**oMN354**	CCGTTCGACTTAGCGTGCGACAACGGTAACGAG
**oMN355**	CTCGTTACCGTTGTCGCACGCTAAGTCGAACGG
**oMN356**	CCGTTCGACTTAGCGATCTGCAACGGTAACGAGGACATT
**oMN357**	AATGTCCTCGTTACCGTTGCAGATCGCTAAGTCGAACGG
**oMN358**	TTCGACTTAGCGATCGACTGCGGTAACGAGGACATTGCT
**oMN359**	AGCAATGTCCTCGTTACCGCAGTCGATCGCTAAGTCGAA
**oMN360**	TTCGACTTAGCGATCGACAACTGCAACGAGGACATTGCTGAAGTG
**oMN361**	CACTTCAGCAATGTCCTCGTTGCAGTTGTCGATCGCTAAGTCGAA
**oMN362**	TTAGCGATCGACAACGGTTGCGAGGACATTGCTGAAGTG
**oMN363**	CACTTCAGCAATGTCCTCGCAACCGTTGTCGATCGCTAA
**oMN364**	GCGATCGACAACGGTAACTGCGACATTGCTGAAGTGCTG
**oMN365**	CAGCACTTCAGCAATGTCGCAGTTACCGTTGTCGATCGC
**oMN366**	ACCCCAAGCTGGCCTCTGAGGCCACCATGGATGGCGCCCCCGCAGG
**oMN367**	CTTGCTCACCATAGATCCACCAGCACTACCACCAGCACTACCACCGGCGGCCGCTGCATTG AGTTCCCTATGCATCTCAC
**oZW25**	ATTATCTAGAATGGTGAGCAAGGGCGAGGAGCTG
**oZW26**	ATTAGAATTCCTACTTGTACAGCTCGTCCATGCCGAGAGTG

Table S2: Oligonucleotides used in this study.

Amino acid sequence of anti-EGFP DARPin **(without cysteine, encoded by pMN43).**
**His-tag, anti-GFP DARPin (3G86.32 C76N), sortase recognition sequence. F152 (hit position for Cys substitution. Note that numbering of the amino acid was done from the N-terminus.**
**MNHKVHHHHHHIEGRHNGSDLGKKLLEAARAGQDDEVRILMANGADVNALDRFGLTPLHLAAQRGHLEIVEVLLK<br>NGADVNAADLWGQTPLHLAATAGHLEIVEVLLKYGADVNALDLIGKTPLHLTAIDGHLEIVEVLLKHGADVNAQD<br>KFGKTAFDISIDNGNEDLAEILQKLNLPETGG***

Amino acid sequence of anti-EpCAM DARPin **(without cysteine, encoded by pMN37).**
**His-tag, anti-EpCAM DARPin (Ec1), sortase recognition sequence. K152 (hit position for Cys substitution. Note that numbering of the amino acid was done from the N-terminus.**
**MNHKVHHHHHHIEGRHNGSDLGKKLLEAARAGQDDEVRILVANGADVNAYFGTTPLHLAAAHGRLEIVEVLLKNG<br>ADVNAQDVWGITPLHLAAYNGHLEIVEVLLKYGADVNAHDTRGWTPLHLAAINGHLEIVEVLLKNVADVNAQDRS<br>GKTPFDLAIDNGNEDIAEVLQKAAKLNLPETGG***

Amino acid sequence of EpCAM-EGFP **(encoded by pMN227)**
**EpCAM, EGFP**
**MAPPQVLAFGLLLAAATATFAAAQEECVCENYKLAVNCFVNNNRQCQCTSVGAQNTVICSKLAAKCLVMKAEMNG<br>SKLGRRAKPEGALQNNDGLYDPDCDESGLFKAKQCNGTSTCWCVNTAGVRRTDKDTEITCSERVRTYWIIIELKH<br>KAREKPYDSKSLRTALQKEITTRYQLDPKFITSILYENNVITIDLVQNSSQKTQNDVDIADVAYYFEKDVKGESL<br>FHSKKMDLTVNGEQLDLDPGQTLIYYVDEKAPEFSMQGLKAGVIAVIVVVVIAVVAGIVVLVISRKKRMAKYEKA<br>EIKEMGEMHRELNAAAAGGSAGGSAGGSMVSKGEELFTGVVPILVELDGDVNGHKFSVSGEGEGDATYGKLTLKF<br>ICTTGKLPVPWPTLVTTLTYGVQCFSRYPDHMKQHDFFKSAMPEGYVQERTIFFKDDGNYKTRAEVKFEGDTLVN<br>RIELKGIDFKEDGNILGHKLEYNYNSHNVYIMADKQKNGIKVNFKIRHNIEDGSVQLADHYQQNTPIGDGPVLLP<br>DNHYLSTQSALSKDPNEKRDHMVLLEFVTAAGITLGMDELYK***

Table S3: Sequences of proteins relevant to this study.

Supporting Figures

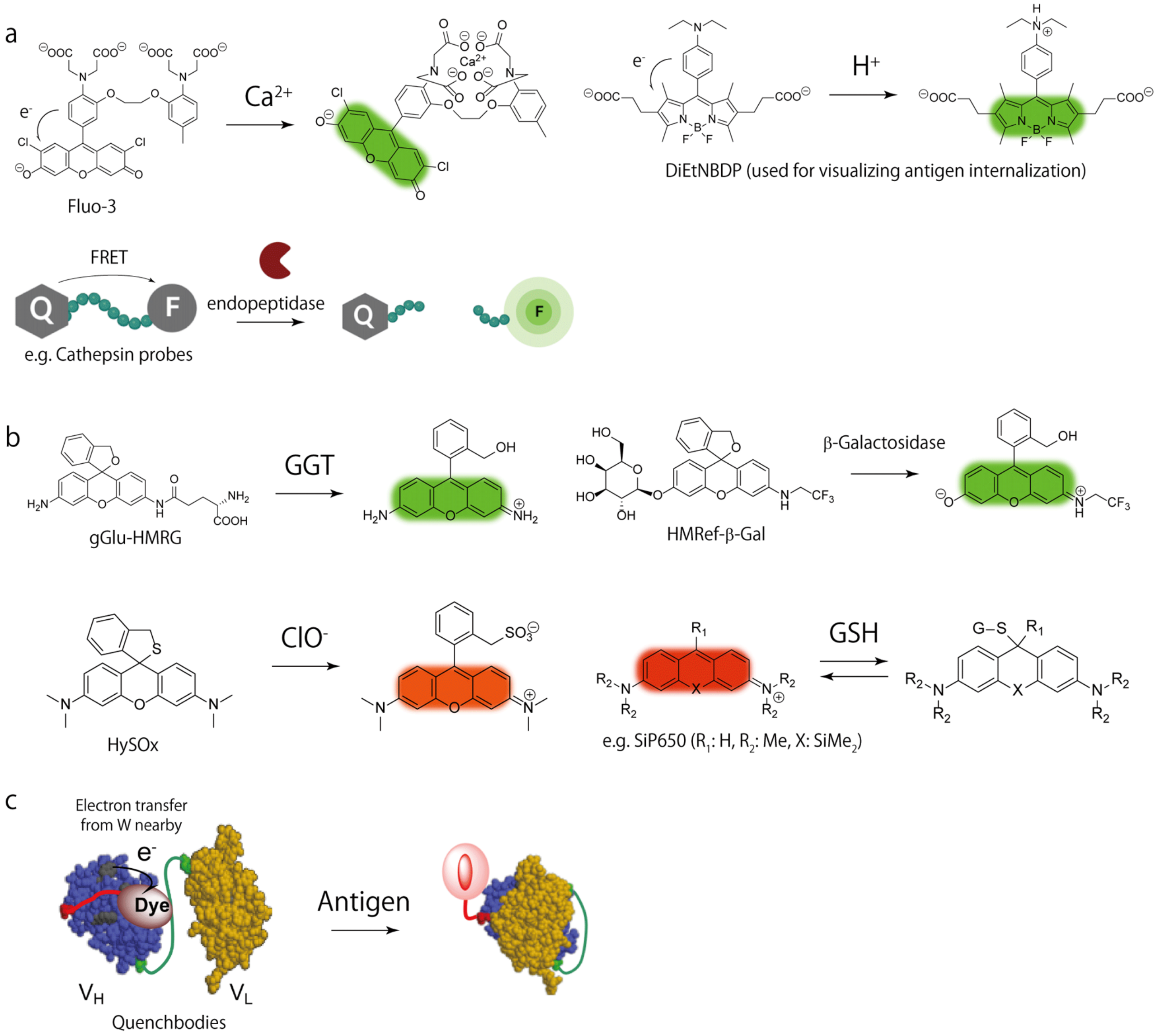

Figure S1. Categorization of existing OFF/ON type fluorogenic probes, with examples. **a**. QY-dependent fluorogenic probes to sense small molecules or enzymes: Fluo-3[16] for sensing Ca^{2+} (QY is controlled by changing the degree of photoinduced electron transfer (PeT) upon Ca^{2+} chelation), DiEtNBDP[17] for sensing intracellular acidic condition after antigen internalization (QY is controlled by changing the degree of PeT upon protonation of the amino group), endopeptidase sensors based on FRET (QY is restored by cleavage of the quencher (FRET acceptor). The figure of endopeptidase probe is adapted from a reference[18] (Available under CC BY 4.0 license. Copyright © 2022, Linders et al). **b**. Absorption-dependent fluorogenic probes to sense small molecules or enzymes: gGlu-HMRG[19,20] for sensing γ-glutamyl transferase (GGT) (the probe is initially colorless and nonfluorescent; colored, highly fluorescent HMRG is generated by the reaction with γ-glutamyl transferase (GGT)). HMRef-β-Gal[21] for sensing β-galactosidase (the probe is initially colorless and nonfluorescent; colored, highly fluorescent HMRef is generated by the reaction with β-galactosidase). GSH probe such as SiP650, which is reversibly quenched by glutathione (GSH)[22] (the probe is colored and highly fluorescent when π-conjugated; when GSH attacks the 9' position of the xanthene moiety, it becomes colorless and nonfluorescent). **c.** QY-dependent fluorogenic probes for antigens: Quenchbodies (typically,

the fluorescence is quenched by PeT from nearby tryptophan in the absence of antigen, but antigen binding releases the quenching. The figure is adapted from a reference[23] (Available under CC BY 4.0 license. Copyright © 2021, Dong and Ueda).

Note regarding probe design and Figure S1

For simplicity, we have focused on OFF/ON-type fluorogenic probes, and have not covered probes based on wavelength shift. So far, no antigen-targeting fluorogenic probes in this category have been reported, though it might be possible to develop such a probe if antigen binding can produce a substantial change in the wavelength of absorption/fluorescence.

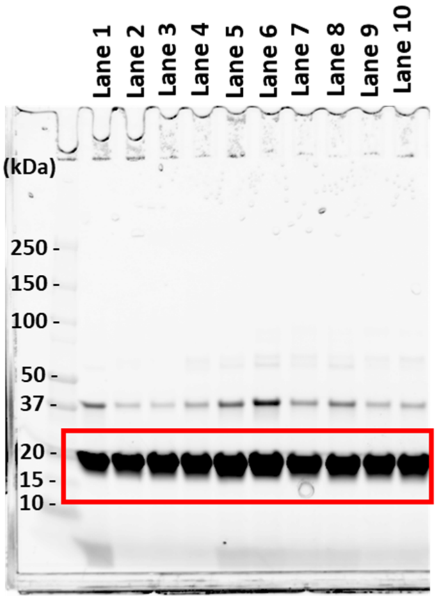

Figure S2: Example of confirmation of sortase-mediated conjugation of 5-Gly-SiP to DARPin 3G86.32 by SiP fluorescence. Lane 1: protein encoded by pMN43 (3G86.32) Lane 2: protein encoded by pMN45 (N147C), Lane 3: protein encoded by pMN46 (A148C) Lane 4: protein encoded by pMN47 (Q149C), Lane 5: protein encoded by pMN49 (K150C), Lane 6: protein encoded by pMN50 (F152C), Lane 7: protein encoded by pMN51 (G152C), Lane 8: protein encoded by pMN52 (K154C), Lane 9: protein encoded by pMN53 (T155C), Lane 10: protein encoded by pMN54 (A156C). Fluorescence was monitored with a LAS4000 using the following settings: Light: red (630 nm Epi), Filter: R670 (Cy5 detection filter).

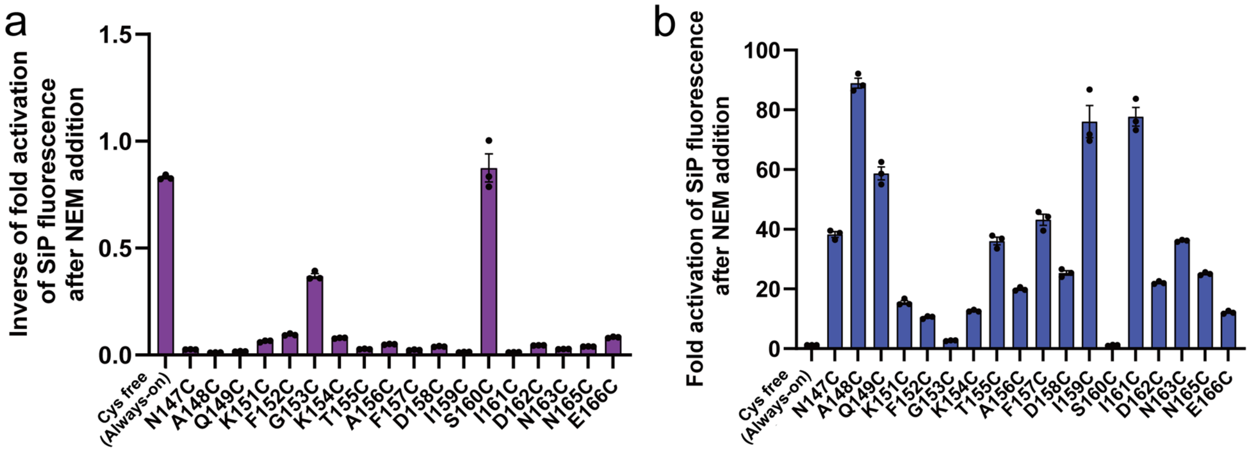

Figure S3: Fold change of SiP fluorescence of the conjugates of 3G86.32 mutants and 5-Gly SiP after the addition of *N*-ethylmaleimide (NEM). Conjugate: 500 nM, NEM: 1 mM. **a.** The inverse of fold activation of SiP fluorescence of each conjugate after NEM addition, which reflects the degree of the initial quenching of the probes in the absence of the target antigen (the lower, the better). **b**. Fold activation of SiP fluorescence of each conjugate after NEM addition. If this value is significantly higher than the fold activation after the addition of GFP (Figure 1c), this indicates that the quenching is not fully relieved upon GFP addition. Error bars represent ±SEM (n = 3). Fluorescence intensity was measured with an Envision plate reader according to the same protocol used for Figure 1c. Excitation: 620 nm (±5 nm), emission: 685 nm (±17.5 nm).

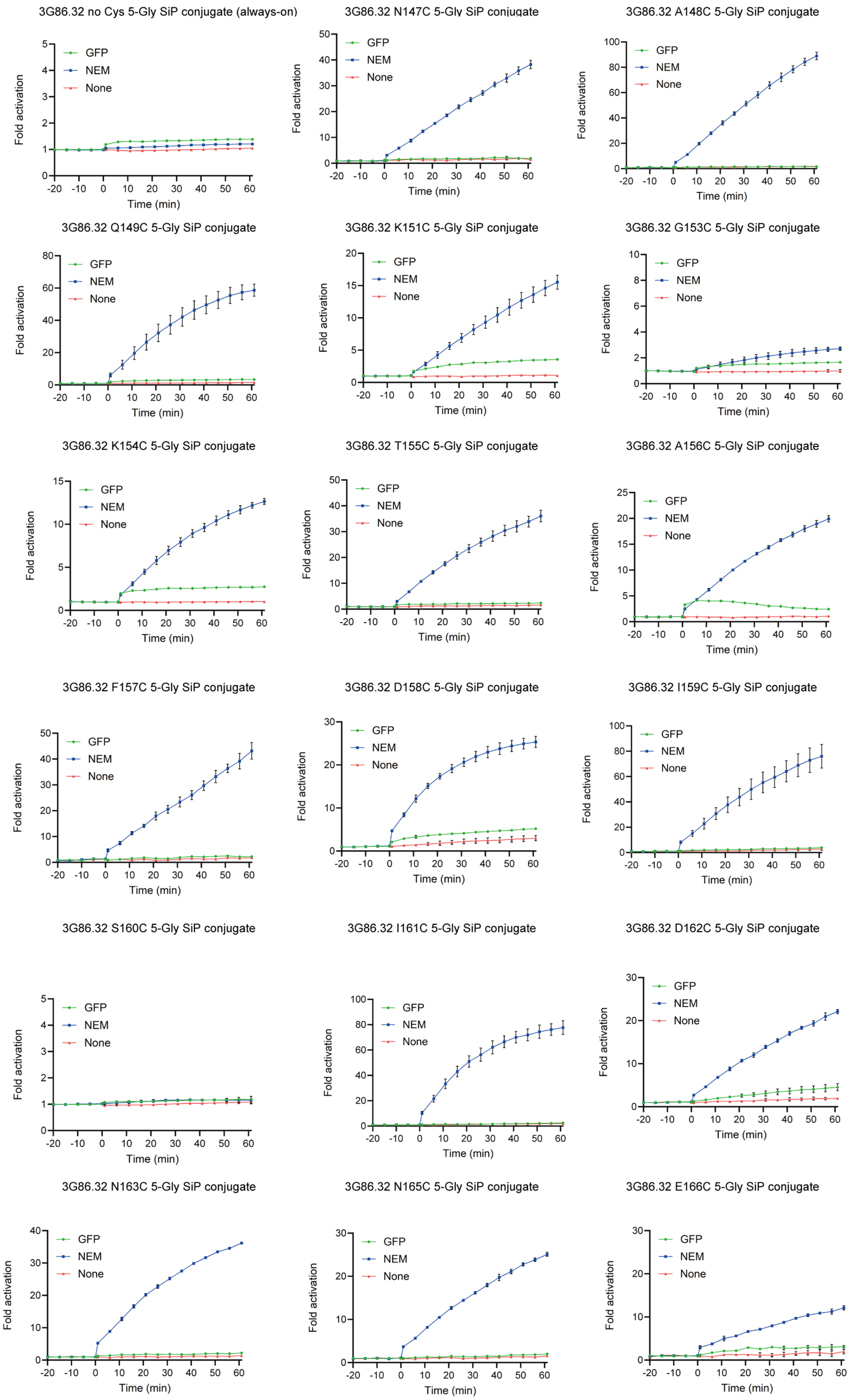
3G86.32 no Cys 5-Gly SiP conjugate (always-on)
3G86.32 N147C 5-Gly SiP conjugate
3G86.32 A148C 5-Gly SiP conjugate
3G86.32 Q149C 5-Gly SiP conjugate
3G86.32 K151C 5-Gly SiP conjugate
3G86.32 G153C 5-Gly SiP conjugate
3G86.32 K154C 5-Gly SiP conjugate
3G86.32 T155C 5-Gly SiP conjugate
3G86.32 A156C 5-Gly SiP conjugate
3G86.32 F157C 5-Gly SiP conjugate
3G86.32 D158C 5-Gly SiP conjugate
3G86.32 I159C 5-Gly SiP conjugate
3G86.32 S160C 5-Gly SiP conjugate
3G86.32 I161C 5-Gly SiP conjugate
3G86.32 D162C 5-Gly SiP conjugate
3G86.32 N163C 5-Gly SiP conjugate
3G86.32 N165C 5-Gly SiP conjugate
3G86.32 E166C 5-Gly SiP conjugate
GFP
NEM
None
Fold activation
Time (min)

Figure S4: Time course of fold change of fluorescence intensity of 500 nM 3G86.32-5-Gly SiP conjugates (other than F152C, shown in Fig 1d) following the addition of GFP (1 μM) or N-ethylmaleimide (NEM) (1 mM). The protocol is the same as for Fig 1d. Error bars represent ±SEM (n = 3). Fluorescence intensity was measured with an Envision plate reader. Excitation: 620 nm (±5 nm), emission: 685 nm (±17.5 nm).

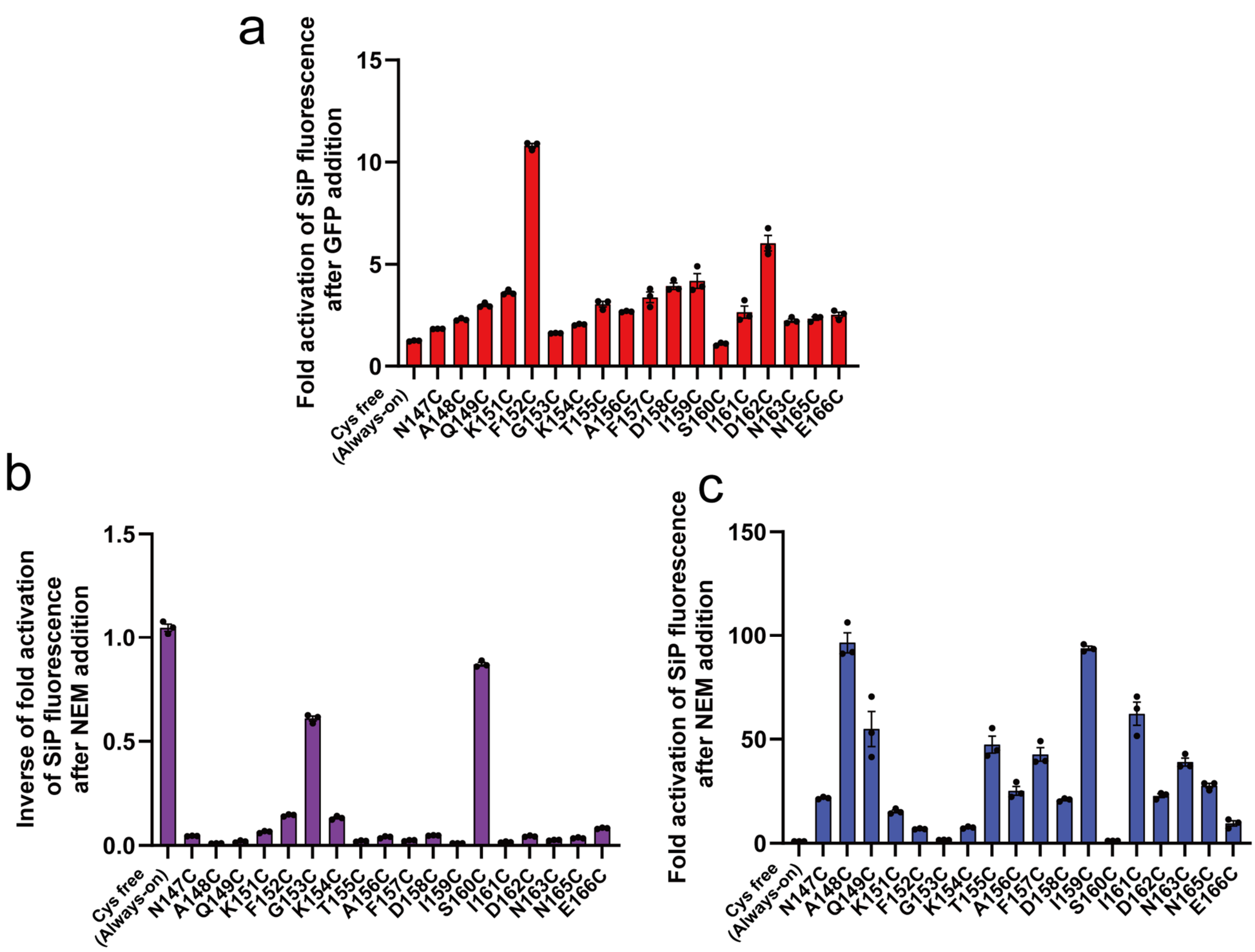

Figure S5: Results of screening of GFP probes constructed with the conjugates of 3G86.32 mutants and 6-Gly SiP. The final concentration of each component in the screening is as follows (the same as Fig 1b,c). Conjugate: 500 nM, GFP or NEM: 1 μM or 1 mM, respectively. **a**. Fold activation of SiP fluorescence of each 6-Gly-SiP conjugate upon GFP addition (the higher, the better). **b**. The inverse of fold activation of SiP fluorescence of each 6-Gly-SiP conjugate after NEM addition, which reflects the degree of the initial quenching of the probes in the absence of the target antigen (the lower, the better). **c**. Fold activation of SiP fluorescence of each conjugate after NEM addition. If the value in **c** is significantly higher than the value in **a**, this means that the quenching is not fully relieved upon GFP addition. Error bars represent ±SEM (n = 3). Fluorescence intensity was measured with an Envision plate reader. Excitation: 620 nm (±5 nm), Emission: 685 nm (±17.5 nm).

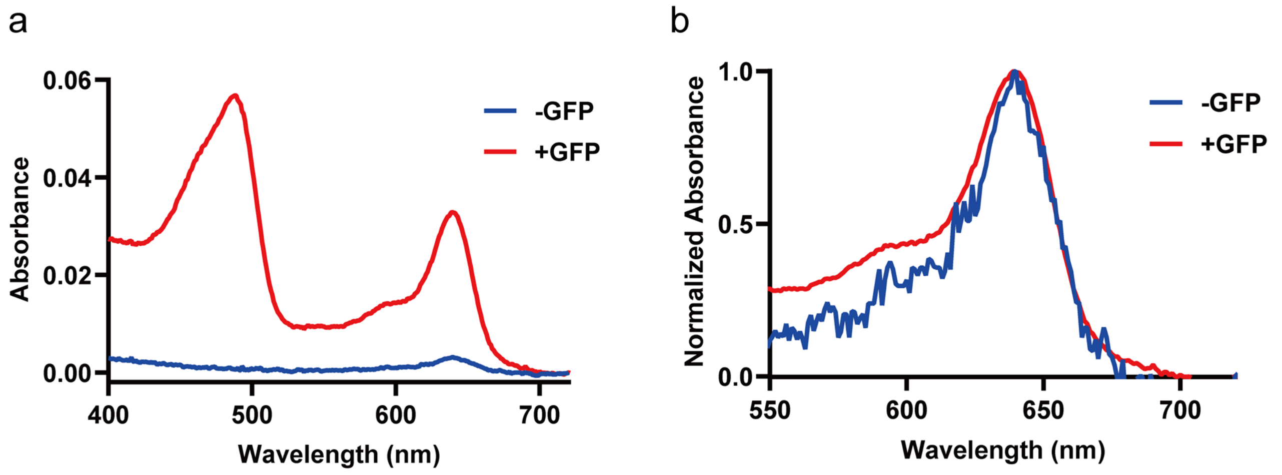

Figure S6: Absorption spectra related to the activatable GFP probe. **a.** Full absorption spectra including the absorption of GFP itself (peak at around 490 nm). Considering the presence of 2 eq. of GFP and the extinction coefficients of GFP (55,900 M^{-1} cm^{-1}) and SiP (assumed to be around 100,000 M^{-1} cm^{-1}), the results suggest that the absorption of SiP is almost fully recovered. **b.** Normalized absorption spectra of SiP of activatable GFP probe before and after the addition of GFP. No significant change in absorption wavelength was observed.

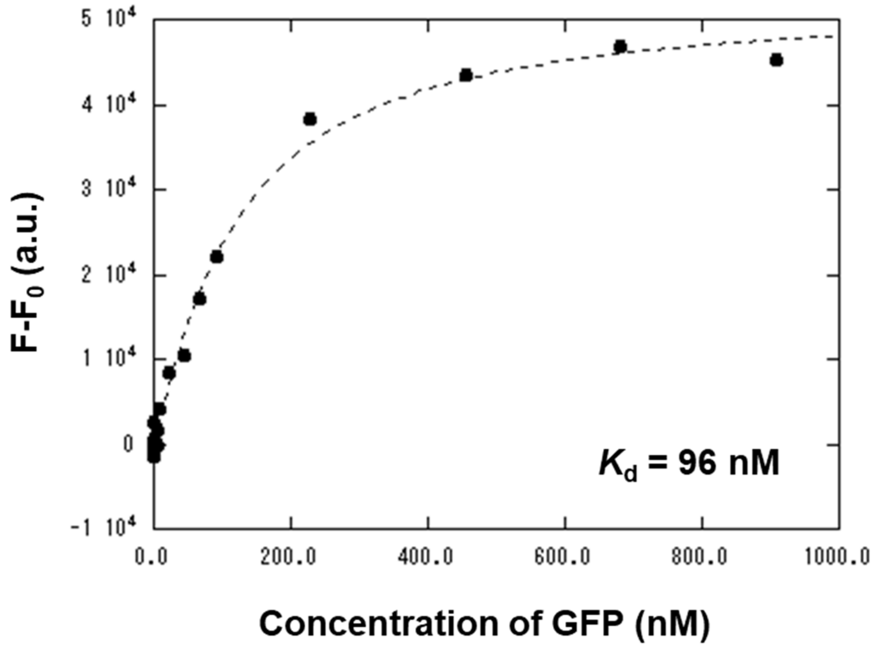

Figure S7: Determination of the dissociation constant of the activatable GFP probe (3G86.32 F152C 5-Gly SiP conjugate) for GFP based on the fluorescence increase. Fitting was conducted as described in the Methods. Fluorescence intensity was measured with an Envision plate reader. Excitation: 620 nm (±5 nm), emission: 685 nm (±17.5 nm).

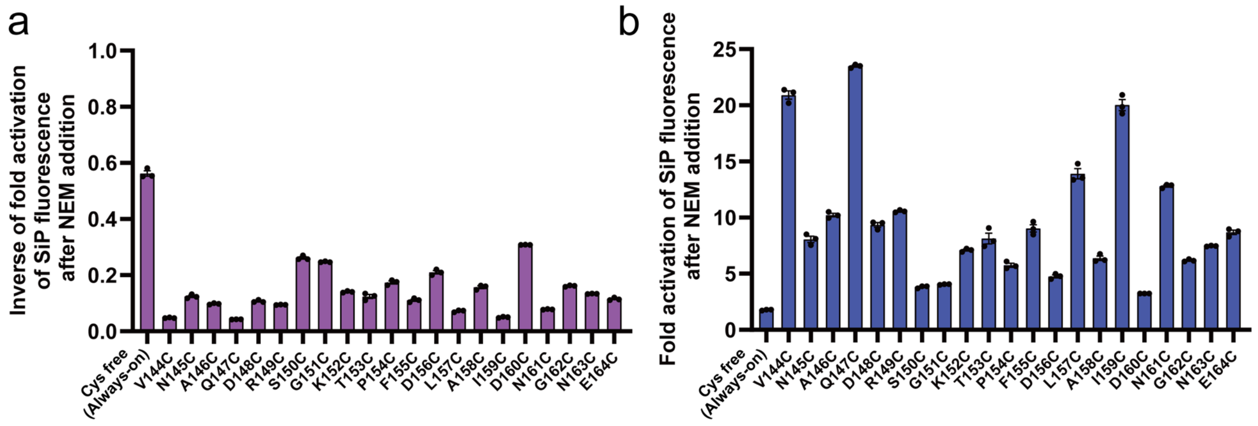

Figure S8: Fold change of SiP fluorescence of the conjugates of Ec1 mutants and 5-Gly SiP after the addition of *N*-ethylmaleimide (NEM). Conjugate: 500 nM, NEM: 1 mM. **a.** The inverse of fold activation of SiP fluorescence of each conjugate after NEM addition, which reflects the degree of the initial quenching of the probe in the absence of the target antigen (the lower, the better). **b**. Fold activation of SiP fluorescence of each conjugate after NEM addition. If this value is significantly higher than the fold activation after the addition of EpCAM ECD (Figure 3b), this means that the quenching is not fully relieved upon EpCAM ECD addition. Error bars represent ±SEM (n = 3). Fluorescence intensity was measured with an Envision plate reader according to the same protocol as used for Figure 3c. Excitation: 620 nm (±5 nm), emission: 685 nm (±17.5 nm).

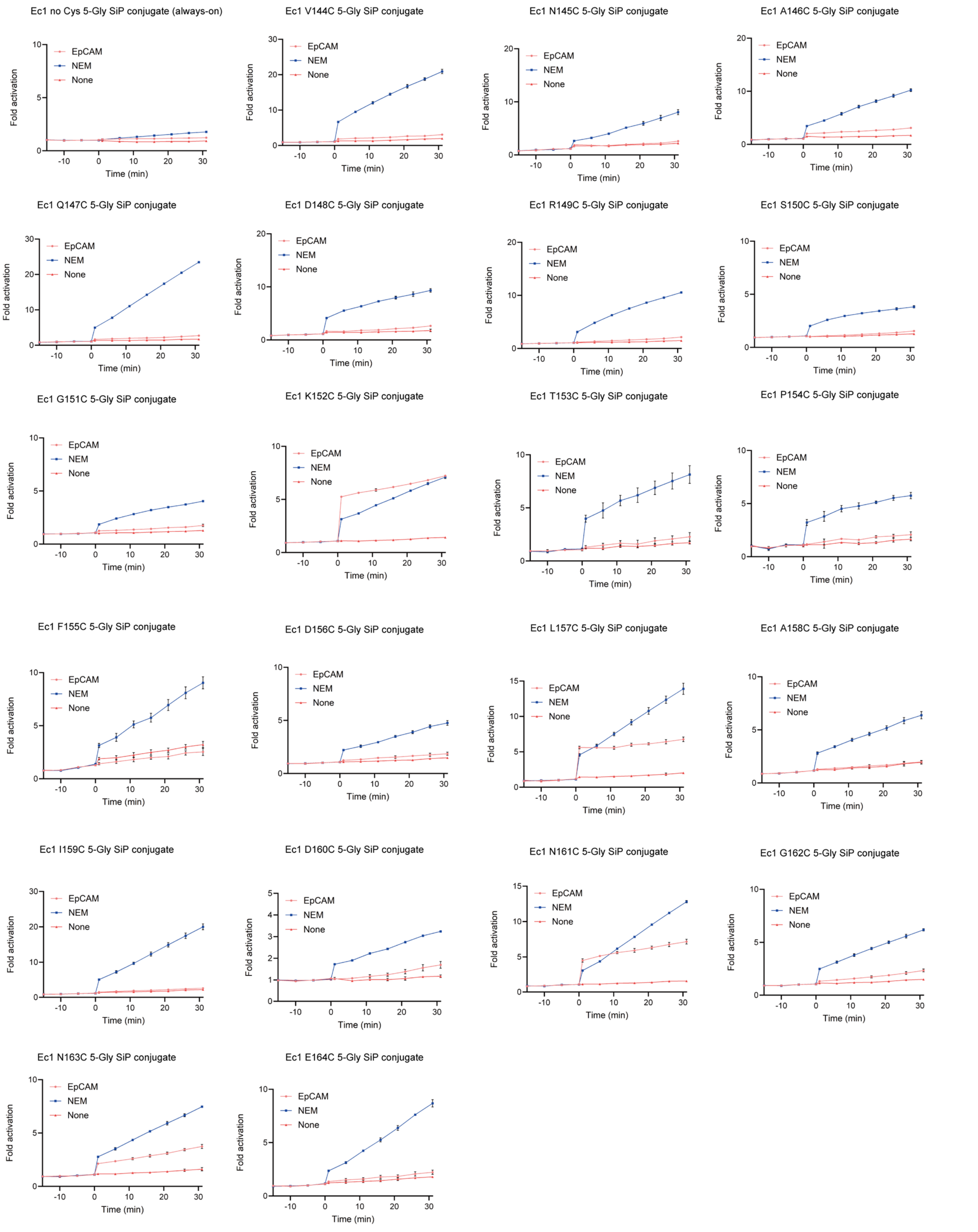

Figure S9: Time-course of fold change of fluorescence intensity of the Ec1 mutants-5-Gly SiP conjugates (500 nM) following the addition of EpCAM ECD (1 μM) or N-ethylmaleimide (NEM) (1 mM). The protocol is the same as for Fig 3c. Error bars represent SEM (n = 3). Note that the conjugate was not pre-treated with DTT for this screening.

Fluorescence intensity was measured with an Envision plate reader. Excitation: 620 nm (±5 nm), emission: 685 nm (±17.5 nm).

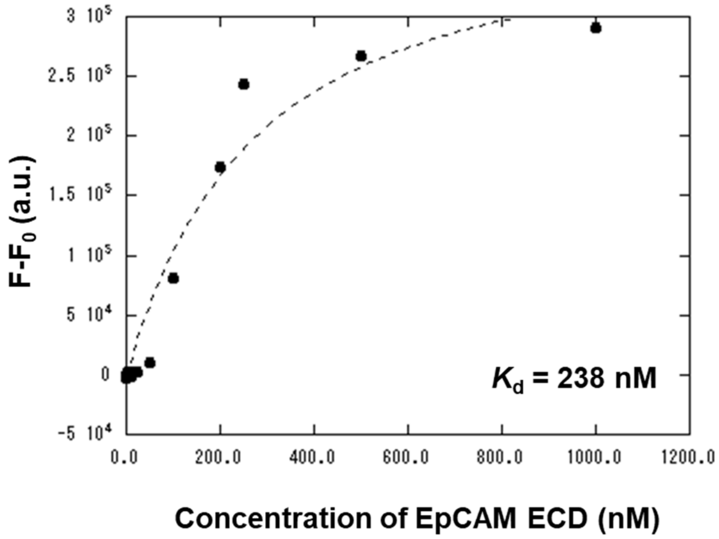

Figure S10: Determination of the dissociation constant of the activatable EpCAM probe (Ec1 K152C 5-Gly SiP conjugate) for EpCAM ECD from the fluorescence increase. Fitting was conducted as described in the Methods. Fluorescence intensity was measured with an Envision plate reader. Excitation: 620 nm (±5 nm), emission: 685 nm (±17.5 nm).

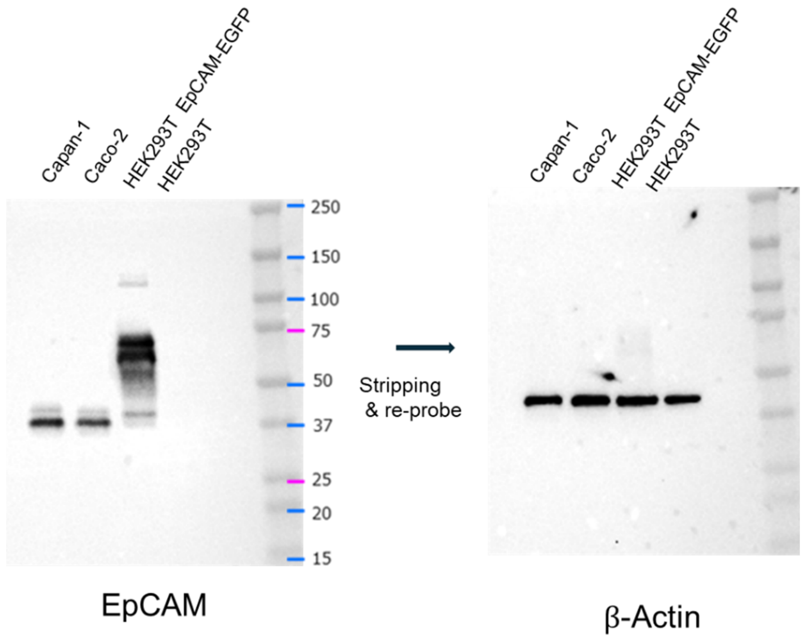

Figure S11: Confirmation of the expression level of EpCAM in the tested cell lines by western blotting. See Methods for the experimental detail. The signal was detected by HRP. The results clearly suggest both Capan-1 cells and Caco-2 cells express EpCAM, while HEK293T cells are originally EpCAM-negative. Note that EGFP is fused to EpCAM for HEK293T cells (the results suggest EpCAM might be glycosylated).

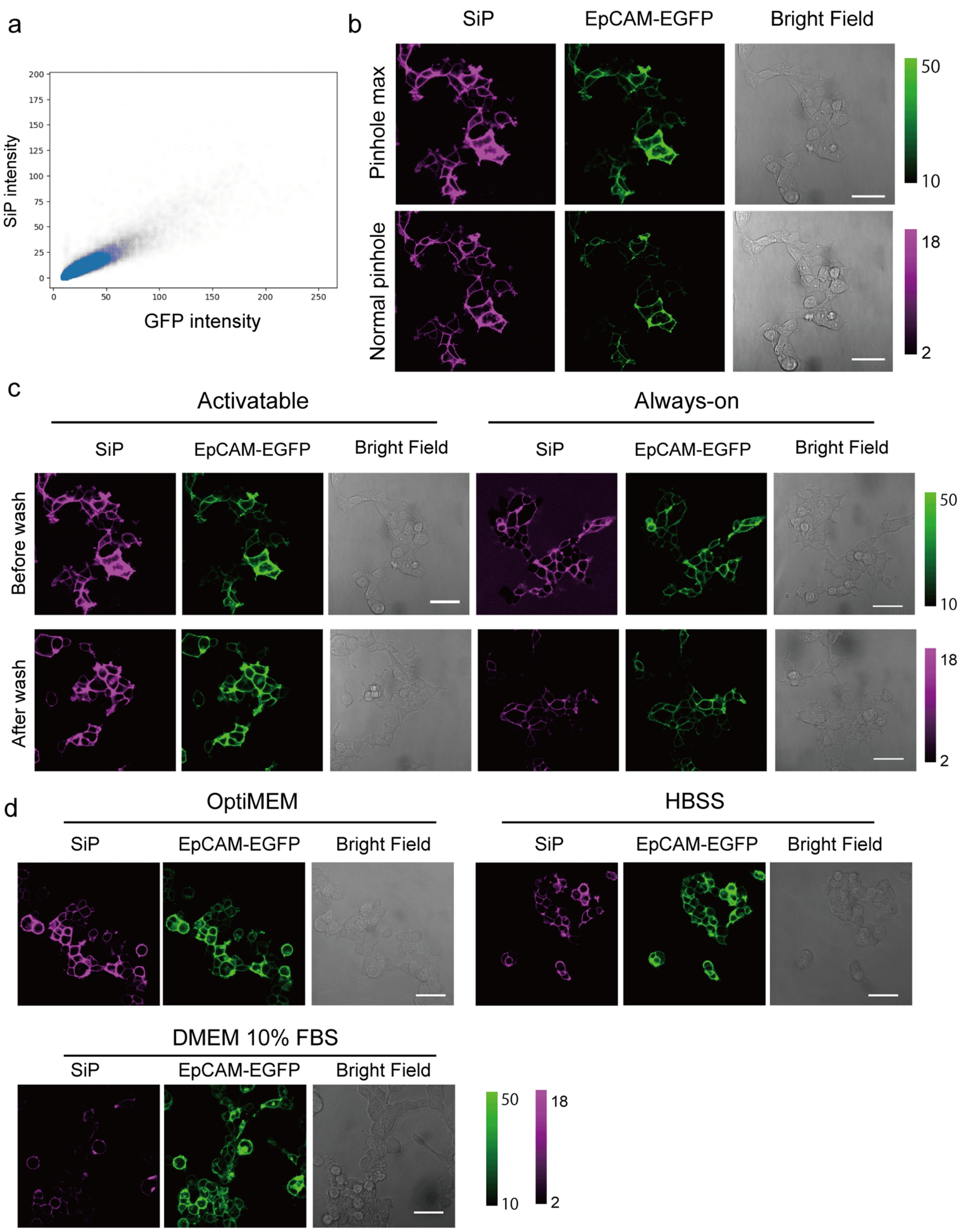

Figure S12: Additional information related to imaging of HEK293T cells expressing EpCAM-EGFP **a**. Correlation between SiP fluorescence and EpCAM-EGFP fluorescence. Pixel-level quantification of SiP and EGFP signals from imaging using the activatable probe (Figure 3d, bottom) revealed a clear correlation between the two. This result

indicates that the developed probe selectively detects EpCAM. **b**. Imaging data acquired using the activatable probe with different pinhole sizes on a confocal microscope. These results show that the signal appearing to originate from inside the cell is due to the use of a large pinhole size during imaging. The top row corresponds to the same images shown in Figure 3d. **c**. Comparison of imaging results before and after washing when using the always-on probe. It is evident that washing removes the extracellular signal, even for the always-on probe. The images of "before wash" are the same as those in Figure 3d. **d**. Imaging of HEK293T EpCAM-EGFP cells with the activatable EpCAM probe in different cell culture media. Probe: 500 nM. Scale bars: 50 μm.

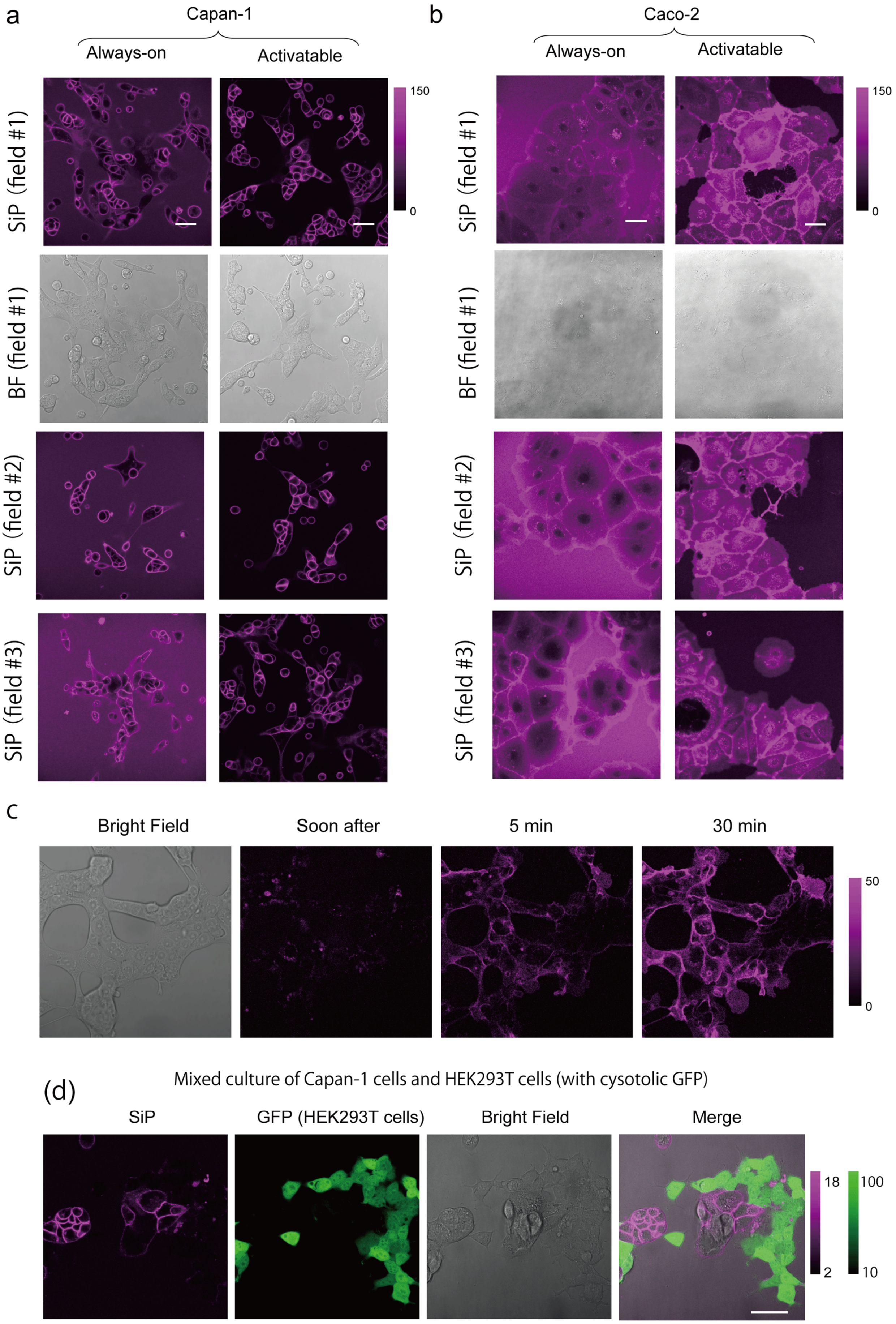
a
Capan-1
Always-on
Activatable
SiP (field #1)
BF (field #1)
SiP (field #2)
SiP (field #3)
150
0
b
Caco-2
Always-on
Activatable
SiP (field #1)
BF (field #1)
SiP (field #2)
SiP (field #3)
150
0
c
Bright Field
Soon after
5 min
30 min
50
0
Mixed culture of Capan-1 cells and HEK293T cells (with cysotolic GFP)
(d)
SiP
GFP (HEK293T cells)
Bright Field
Merge
18
2
100
10

Figure S13: Additional information related to imaging of Capan-1 and Caco-2 cells **a**. Multiple images of Capan-1 cells with the EpCAM probes. The images of field #1 are the same as those in Figure 3e, and bright-field images are also shown for these fields. **b.** Multiple images of Caco-2 cells with the EpCAM probes. The images of field #1 are the same as those in Figure 3f, and bright-field images are also shown for these fields. **c.** Fast live-cell imaging of Capan-1 cells with the activatable EpCAM probe under a wash-free condition. "Soon after" means within a few tens of seconds. **d.** Live cell imaging of a mixed co-culture of EpCAM-positive Capan-1 cells and EpCAM-negative HEK293T cells (expressing cytosolic GFP as a marker) with the activatable probe. For **d**,**e**,**f**,**h**, all the data was obtained with 500 nM probe in DPBS. The images were obtained with a Leica TCS SP5 within 30 min after the addition of the probe, with a maximum pinhole size in order to emphasize the background (see Figure S12b for this effect). Ex: 633 nm, Em: 653-750 nm for SiP fluorescence (shown in magenta), and Ex: 488 nm, Em: 500-600 nm for GFP fluorescence. Note that the look-up tables cannot be directly compared between each subfigure due to the difference in the microscope settings.

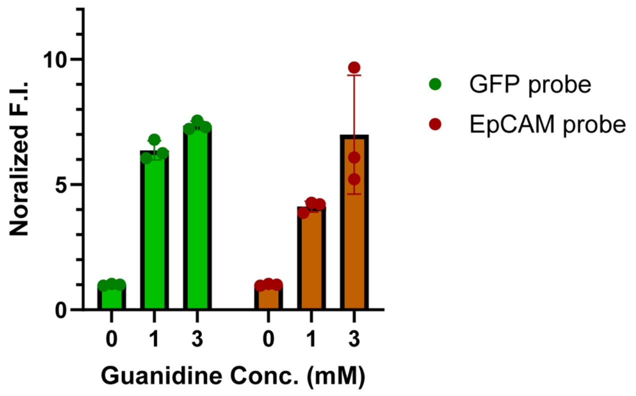

Figure S14: Fluorescence change upon treating the probes with various concentrations of guanidine. 500 nM activatable GFP/EpCAM probe in 10 mM HEPES buffer (pH 7.4) was supplemented with the indicated concentrations of guanidine hydrochloride, and the fluorescence intensity was measured with an Envision plate reader. Excitation: 620 nm (±5 nm), emission: 685 nm (±17.5 nm). The signal intensity was normalized to the value without guanidine.

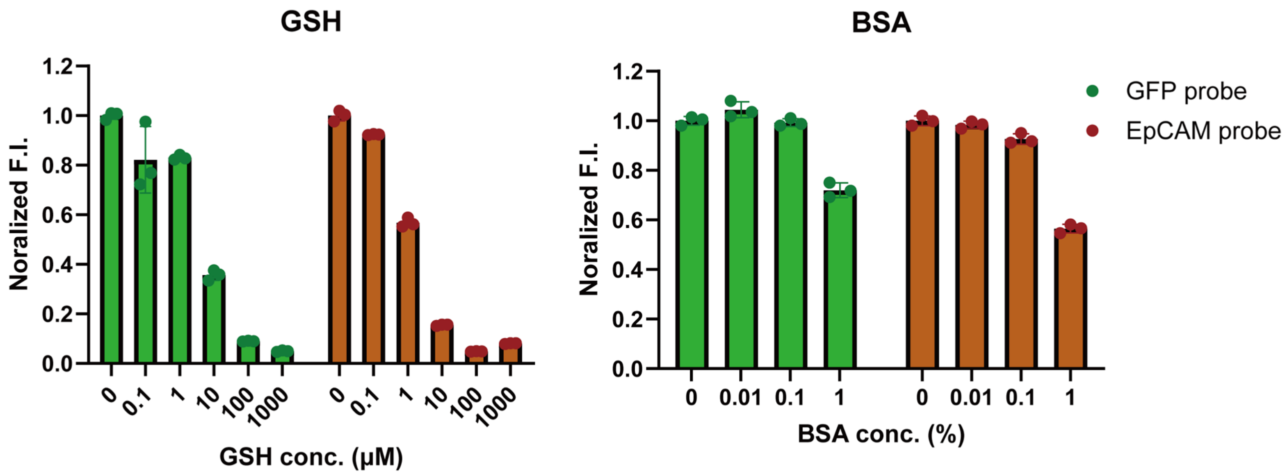

Figure S15 : Susceptibility of the probes to various concentrations of glutathione (GSH) and bovine serum albumin (BSA). The always-on GFP or EpCAM probe was treated with various concentrations of GSH and BSA, and the fluorescence intensity was measured with an Envision plate reader (normalized to the fluorescence intensity without additives).

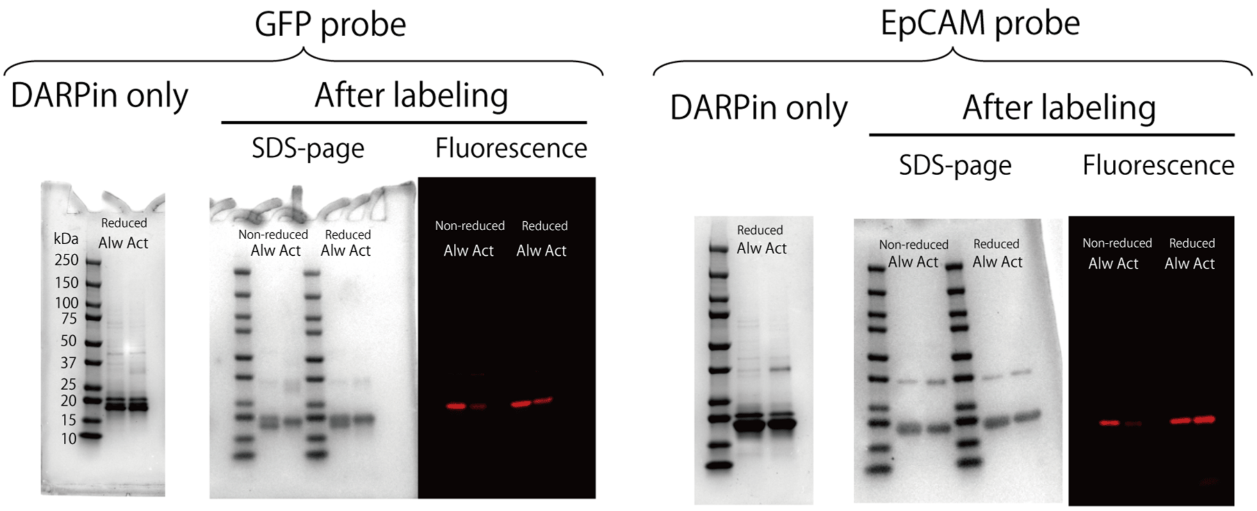

Figure S16: Quality check of the purified DARPin and the probes. Alw: Always-on probe. Act: Activatable probe. Reduced: Samples were treated with SDS-PAGE sample buffer (containing 2-mercaptoethanol; this could react with SiP but fluorescence can be observed after SDS-PAGE because the reaction is reversible) and boiled at 95°C 10 min. Non-reduced: Samples were directly subjected to SDS-PAGE without treatment. 'DARPin only' means the SDS-PAGE analysis of recombinant DARPin after His-tag-based purification. 'After labeling' means the analysis of DARPin-SiP conjugates. Fluorescence was monitored with an iBright FL1500 (Ex: 610-660 nm, Em: 710-730 nm). For non-reduced samples, partial quenching of SiP can still be observed.

Methods for organic synthesis

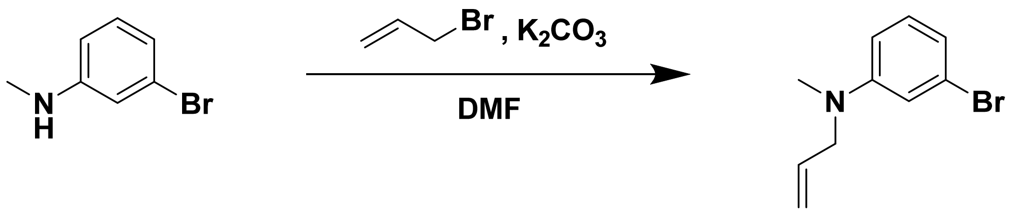

1

Scheme S1: Synthesis of compound 1

Compound **1** was synthesized as described previously[22]. Yield 93%.

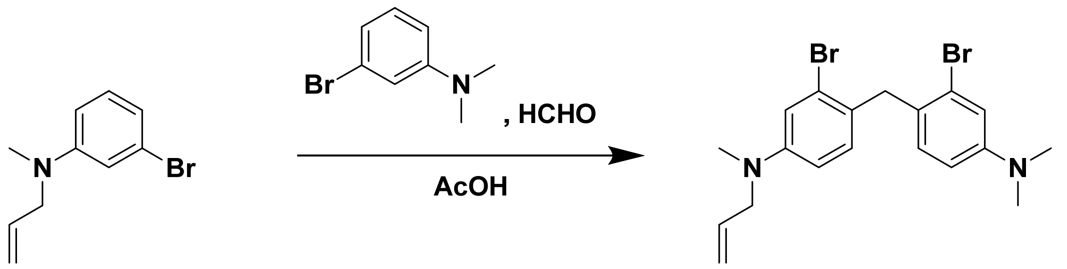

1

2

Scheme S2: Synthesis of compound 2

Compound **2** was synthesized as described previously[22]. Yield 20%.

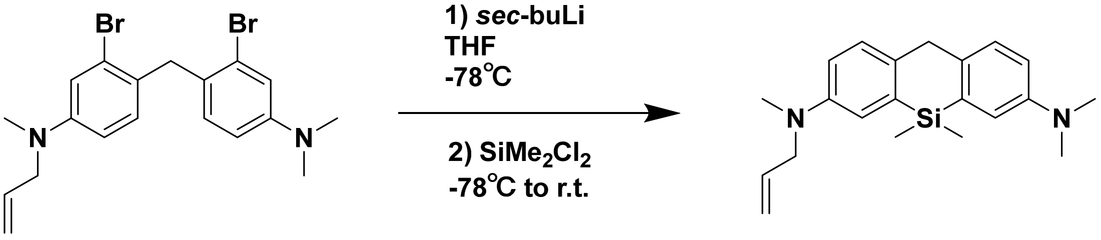

2

3

Scheme S3: Synthesis of compound 3

Compound **3** was synthesized as described previously[22]. Yield 78%.

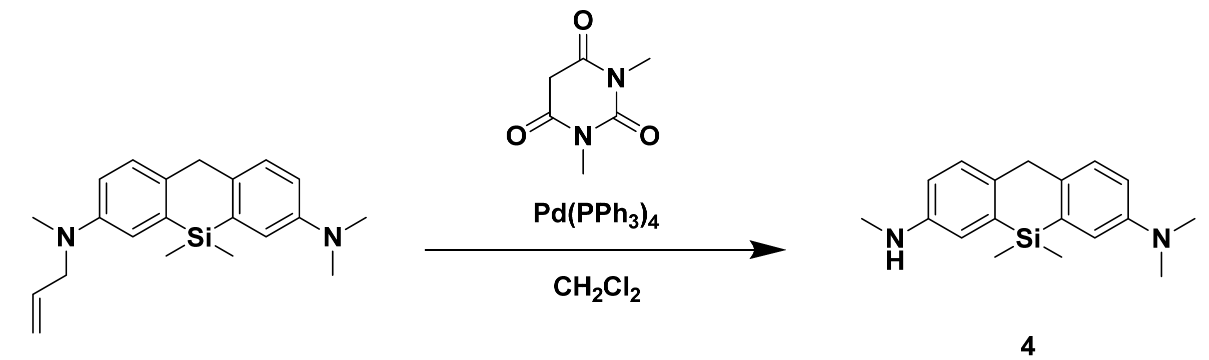

3 4

Scheme S4: Synthesis of compound 3

Compound **4** was synthesized as described previously[22]. Yield 85%.

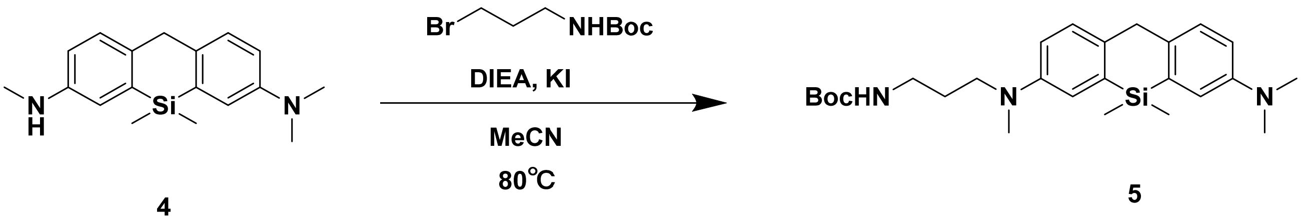

4 5

Scheme S5: Synthesis of compound 5

To a solution of **4** (234 mg, 0.791 mmol, 1 eq) in acetonitrile (20 ml) in a two-necked flask were added 3-Boc amino propyl bromide (752 mg, 3.16 mmol, 4 eq), DIEA (408 mg, 548 l, 3.16 mmol, 4 eq) and potassium iodide (catalytic amount). The mixture was stirred at 80 °C for 4 h. After cooling to room temperature, water (2 ml) was added, followed by dichloromethane. The organic phase was collected, washed with water and brine, dried over Na_2SO_4 filtered, and evaporated. The residue was purified by flash column chromatography (n-hexane: AcOEt = 100:0 to 50:50) to afford compound **5** (63.6 mg, 0.140 mmol, yield 18 %). ^{1}H NMR (400 MHz, $CDCl_3$): δ 0.50 (s, 6H), 1.48 (s, 9H), 1.79 (quin, 2H, J = 7.0 Hz), 2.94 (s, 3H), 2.96 (s, 6H), 3.20 (br q, 2H, J = 6.0 Hz), 3.38 (t, 2H, J = 7.1 Hz), 3.99 (s, 2H), 4.68 (br, 1H), 6.72 (dd, 1H, J = 2.8 Hz, 8.4 Hz), 6.77 (dd, 1H, J = 2.8 Hz, 8.3 Hz), 6.99 (d, 1H, J = 2.8 Hz), 7.04 (d, 1H, J = 2.8 Hz), 7.21 (d, 1H, J = 8.4 Hz), 7.22 (d, 1H, J = 8.4 Hz); ^{13}C NMR (101 MHz, $CDCl_3$): δ -2.7, 27.3, 28.5, 38.8, 39.2, 41.2, 50.7, 79.2, 113.9, 114.2, 117.4, 117.6, 128.5, 128.7, 134.9, 135.2, 136.0, 136.3, 147.3, 148.7, 156.1; HRMS (ESI$^+$): Calcd for [M+H$^+$], 454.28843, Found, 454.28842.

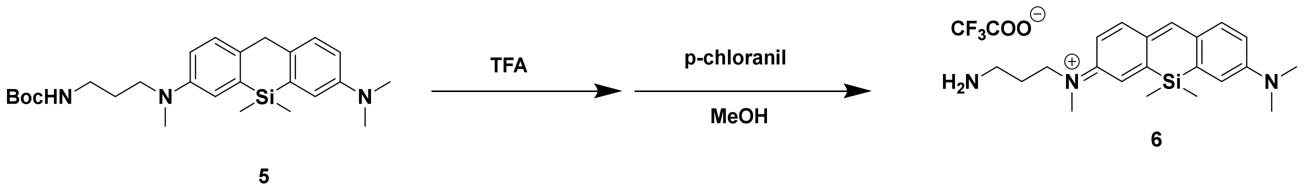

Scheme S6: Synthesis of compound 6

TFA 1 ml was added to **5** (264 mg, 0.58 mmol) on ice and the mixture was stirred at room temperature for 30 minutes. TFA was removed using an evaporator to give a residue. To the solution of the residue in 10 ml of MeOH in a flask was added p-chloranil (159 mg, 0.64 mmol). The mixture was stirred at r. t. and MeOH was removed in a rotary evaporator. The residue was purified by reverse-phase liquid chromatography with an Isolera (H_2O with 0.1 % TFA: MeCN with 1 % H_2O = 90:10 to 0:100). The fractions containing the target product were collected and evaporated to afford compound **6** as TFA salt (103.1 mg, yield 38 %). ^{1}H NMR (400 MHz, CD_3OD): δ 0.54 (s, 6H), 2.07 (quin, 2H, J = 7.7 Hz), 3.04-3.08 (m, 2H), 3.34 (s, 3H), 3.40 (s, 6H), 3.81 (t, 2H, J = 8.0 Hz), 6.98-7.04 (m, 2H), 7.32 (d, 1H, J = 2.4 Hz), 7.36 (d, 1H, J = 2.4 Hz), 7.72 (d, 1H, J = 2.4 Hz), 7.75 (d, 1H, J = 2.4 Hz), 7.90 (s, 1H); ^{13}C NMR (101 MHz, $CDCl_3$, containing peaks from TFA): δ -1.4, 26.6, 38.2, 39.5, 41.2, 50.7, 115.3 (q, J = 4.0 Hz, from TFA), 118.8, 121.8, 122.6, 129.1, 129.1, 144.3, 145.1, 148.8, 149.7, 155.8, 157.2, 161.1, 161.4 ; HRMS (ESI+): Calcd for [M^+], 352.22035, Found, 352.22035.

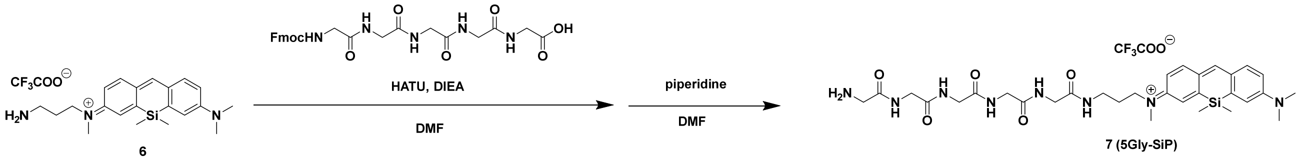

Scheme S7: Synthesis of compound 7 (5-Gly-SiP)

To compound **6** (34 mg, 0.096 mmol) in 3 mL DMF were added HATU (73 mg, 0.192 mmol, 2 eq), DIEA (12 mg, 0.096 mmol, 1 eq), and Fmoc-Gly-Gly-Gly-Gly-Gly-OH (101 mg, 0.192 mmol, 2 eq, synthesized by Genscript). The mixture was stirred at room temperature for 14 hours, then the solvent was evaporated, and the residue was purified by HPLC (H_2O with 0.1 % TFA and 1 % MeCN : MeCN with 1 % H_2O = 90:10 to 10:90 for 40 min). The eluate was evaporated and the residue was dissolved in 2 ml of 40% piperidine in DMF. The solution was stirred at room temperature for 1 hour to deprotect the Fmoc group, then evaporated. The residue was purified by HPLC (H_2O with 0.1 % TFA and 1 % MeCN : MeCN with 1 % H_2O = 90:10 to 10:90 for 40 min). The collected fractions were evaporated and lyophilized to obtain compound **7** as TFA salt (14.9 mg, 2 steps yield 24 %). HRMS (ESI$^+$): Calcd for [M^+], 637.32767, Found, 637.32730.

Analytical HPLC of compound 7

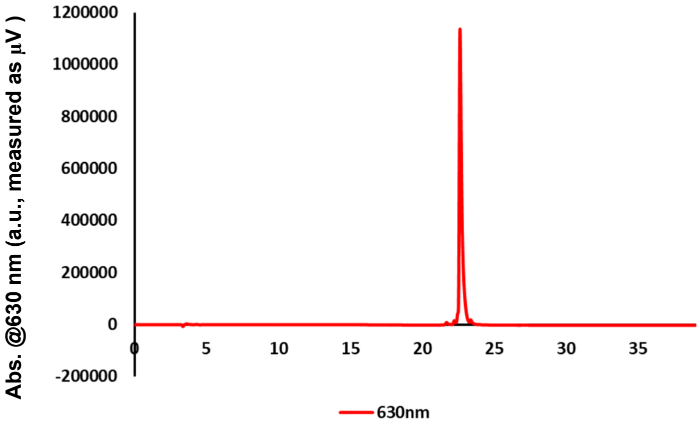

Analysis condition
Column: Inertsil ODS-3 4.6 mm × 250 mm
Eluent: A) H_2O with 0.1 % TFA B) MeCN with 1 % H_2O
Gradient: A:B = 90:10 to 10:90 for 40 min
Flow rate: 1 ml/min

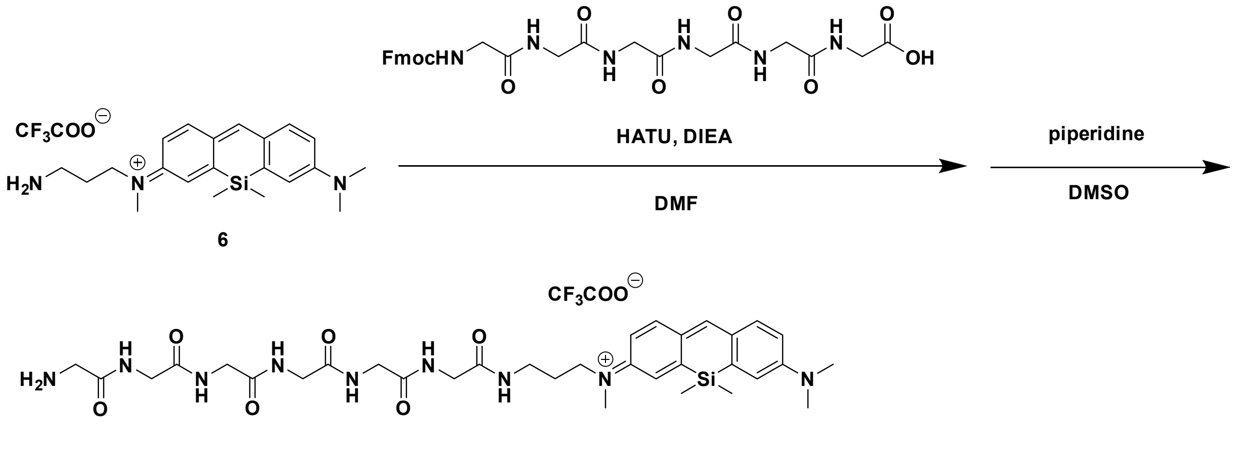

8 (6Gly-SiP)

Scheme S8: Synthesis of compound 8 (6-Gly-SiP)

To compound **6** (3.9 mg, 0.011 mmol) in DMF were added HATU (16 mg, 0.044 mmol, 4 eq), DIEA (5.7 mg, 0.044 mmol, 1 eq), and Fmoc-Gly-Gly-Gly-Gly-Gly-Gly-OH (crude product of solid-phase synthesis using Syro I (Biotage), more than 5 eq). The mixture was stirred at room temperature for 30 mins. The solvent was evaporated, and the residue was purified by HPLC (H_2O with 0.1 % TFA and 1 % MeCN: MeCN with 1 % H_2O = 90:10 to 10:90 for 40 min). The eluate was evaporated and the residue was dissolved in 200 µl of 50 % piperidine in DMSO. This solution was stirred at room temperature for 10 mins to deprotect the Fmoc. The solvent was evaporated and the residue was purified by HPLC (H_2O with 0.1 % TFA and 1 % MeCN: MeCN with 1 % H_2O = 90:10 to 10:90 for 40 min). The acetonitrile was removed from the collected fractions in a rotary evaporator and the residue was lyophilized to obtain compound **8** as TFA salt (y. trace). HRMS (ESI^+): Calcd for $[M^+]$, 694.34913, Found, 694.34925. Concentration of the 6-SiP-Gly was calculated by assuming an extinction co-efficient of 100,000 M^{-1} cm^{-1} at the λ_{max} as described in the Method section.

Analytical HPLC of compound 8

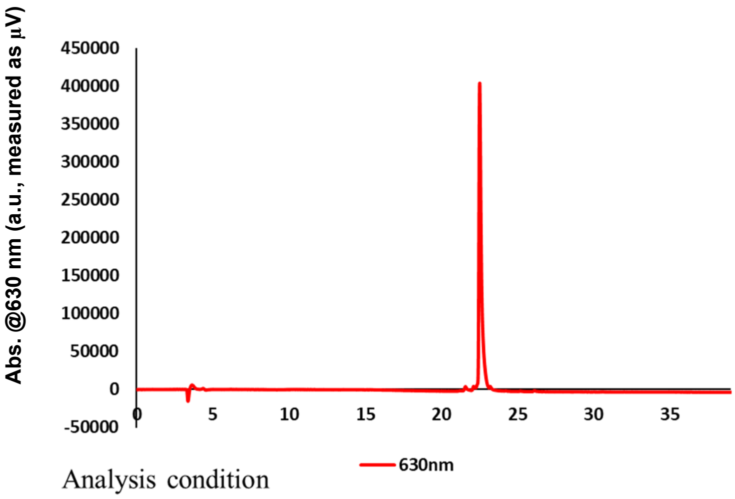

Analysis condition
Column: Inertsil ODS-3 4.6 mm × 250 mm
Eluent: A) H_2O with 0.1 % TFA B) MeCN with 1 % H_2O
Gradient: A:B = 90:10 to 10:90 for 40 min
Flow rate: 1 ml/min

NMR charts:

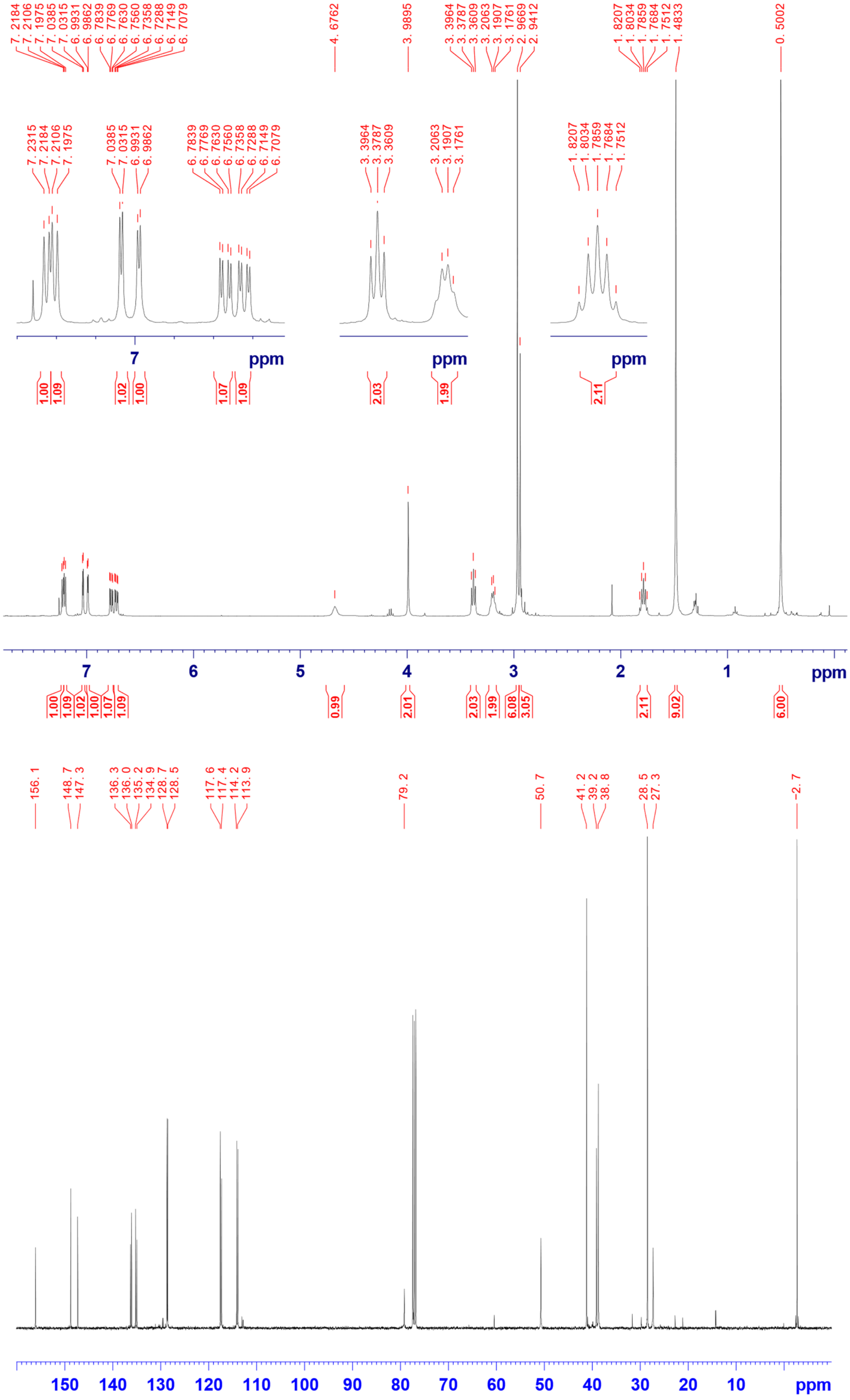

Chart S1: NMR spectra of compound 5 in $CDCl_3$. Top: ^{1}H NMR, Bottom: ^{13}C NMR.

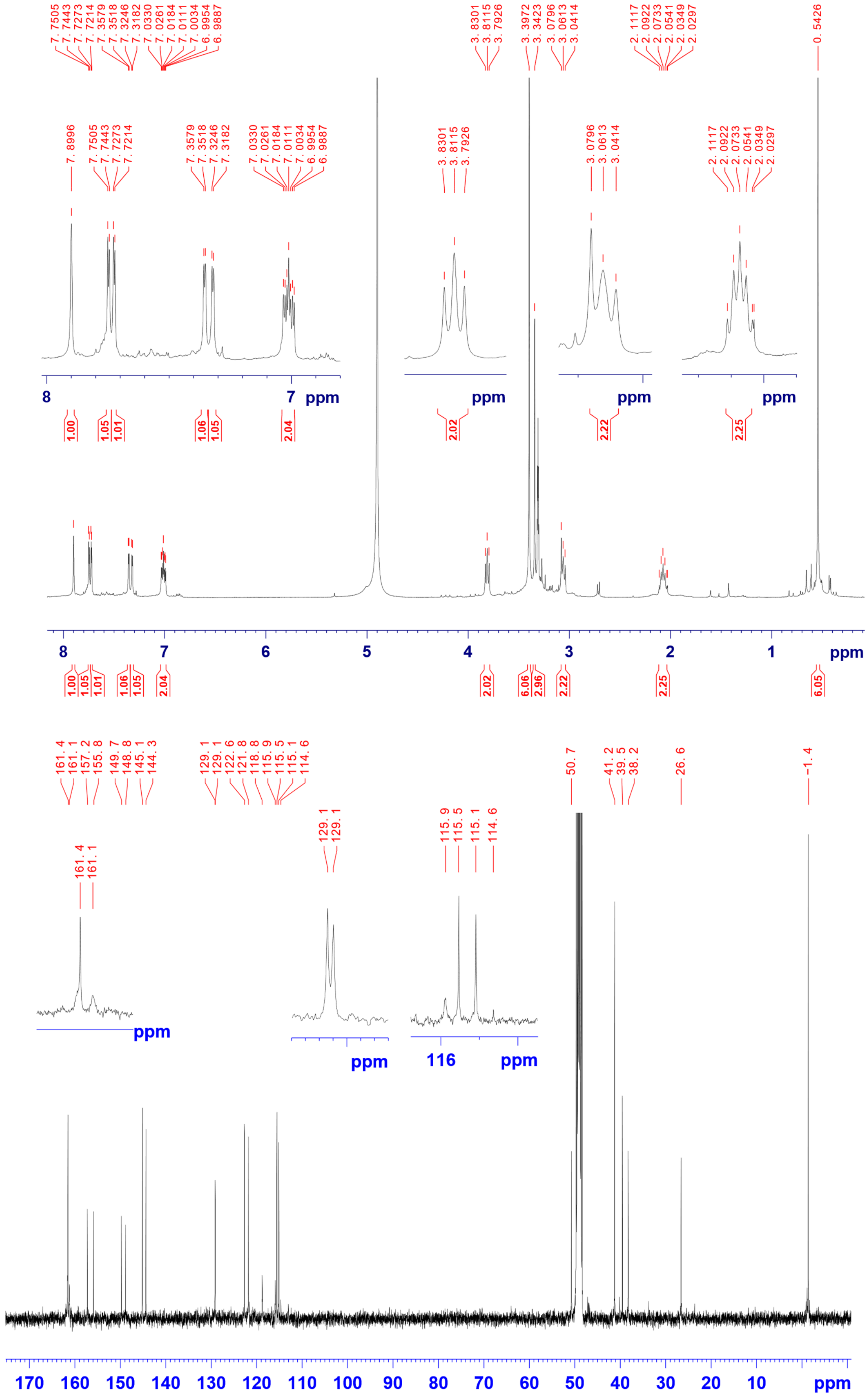

Chart S2: NMR spectra of compound 6 (TFA salt) in CD_3OD. Top: ^1H NMR, Bottom: ^{13}C NMR.

Supporting references

1. Edelheit, O., Hanukoglu, A. & Hanukoglu, I. Simple and efficient site-directed mutagenesis using two single-primer reactions in parallel to generate mutants for protein structure-function studies. *BMC Biotechnol.* **9**, 61 (2009).
2. Yao, Q., Weaver, S. J., Mock, J.-Y. & Jensen, G. J. Fusion of DARPin to Aldolase Enables Visualization of Small Protein by Cryo-EM. *Structure* **27**, 1148-1155.e3 (2019).
3. Bekker, H. *et. al.* A. Gromacs: A parallel computer for molecular dynamics simulations. *Physics Computing '92* (ed. RA DeGroot, J. N.), 1993, 252–256.
4. Stroet, M. *et al.* Automated Topology Builder Version 3.0: Prediction of Solvation Free Enthalpies in Water and Hexane. *J. Chem. Theory Comput.* **14**, 5834–5845 (2018).
5. Lemkul, J. A. & Bevan, D. R. Assessing the stability of Alzheimer's amyloid protofibrils using molecular dynamics. *J. Phys. Chem. B* **114**, 1652–1660 (2010).
6. Jurrus, E. *et al.* Improvements to the APBS biomolecular solvation software suite. *Protein Sci.* **27**, 112–128 (2018).
7. Frisch, M. J. *et al. Gaussian 16 Rev. C.01*. (Wallingford, CT, 2016).
8. Jarmoskaite, I., AlSadhan, I., Vaidyanathan, P. P. & Herschlag, D. How to measure and evaluate binding affinities. *Elife* **9**, e57264 (2020).
9. Chen, I., Dorr, B. M. & Liu, D. R. A general strategy for the evolution of bond-forming enzymes using yeast display. *Proc. Natl. Acad. Sci. U. S. A.* **108**, 11399–11404 (2011).
10. Kowarz, E., Löscher, D. & Marschalek, R. Optimized Sleeping Beauty transposons rapidly generate stable transgenic cell lines. *Biotechnol. J.* **10**, 647–653 (2015).
11. Mátés, L. *et al.* Molecular evolution of a novel hyperactive Sleeping Beauty transposase enables robust stable gene transfer in vertebrates. *Nat. Genet.* **41**, 753–761 (2009).
12. Stefan, N. *et al.* DARPins recognizing the tumor-associated antigen EpCAM selected by phage and ribosome display and engineered for multivalency. *J. Mol. Biol.* **413**, 826–843 (2011).
13. Kojima, R., Scheller, L. & Fussenegger, M. Nonimmune cells equipped with T-cell-receptor-like signaling for cancer cell ablation. *Nat. Chem. Biol.* **14**, 42–49 (2018).
14. Zahnd, C. *et al.* A designed ankyrin repeat protein evolved to picomolar affinity to Her2. *J. Mol. Biol.* **369**, 1015–1028 (2007).
15. Brauchle, M. *et al.* Protein interference applications in cellular and developmental biology using DARPins that recognize GFP and mCherry. *Biol. Open* **3**, 1252–1261 (2014).
16. Kao, J. P., Harootunian, A. T. & Tsien, R. Y. Photochemically generated cytosolic calcium pulses and their detection by fluo-3. *J. Biol. Chem.* **264**, 8179–8184 (1989).
17. Urano, Y. *et al.* Selective molecular imaging of viable cancer cells with pH-activatable fluorescence probes. *Nat. Med.* **15**, 104–109 (2009).
18. Linders, D. G. J. *et al.* Cysteine cathepsins in breast cancer: Promising targets for fluorescence-guided surgery. *Mol. Imaging Biol.* **25**, 58–73 (2023).
19. Urano, Y. *et al.* Rapid cancer detection by topically spraying a γ-glutamyltranspeptidase-activated fluorescent probe. *Sci. Transl. Med.* **3**, 110ra119 (2011).
20. Sakabe, M. *et al.* Rational design of highly sensitive fluorescence probes for protease and glycosidase based on precisely controlled spirocyclization. *J. Am. Chem. Soc.* **135**, 409–414 (2013).
21. Asanuma, D. *et al.* Sensitive β-galactosidase-targeting fluorescence probe for visualizing small peritoneal metastatic tumours *in vivo*. *Nat. Commun.* **6**, 6493, (2015).
22. Morozumi, A. *et al.* Spontaneously Blinking Fluorophores Based on Nucleophilic Addition/Dissociation of Intracellular Glutathione for Live-Cell Super-resolution Imaging. *J. Am. Chem. Soc.* **142**, 9625–9633 (2020).
23. Dong, J. & Ueda, H. Recent Advances in Quenchbody, a Fluorescent Immunosensor. *Sensors* **21**, 1223, (2021).
