# Supplementary material for: Design of Antigen-Targeting Fluorogenic Probes Utilizing Intramolecular Addition Reaction of Protein-Dye Hybrids
Source: J Am Chem Soc. 2025 Jul 23;147(34):30684–93. doi: 10.1021/jacs.5c04193 (PMC12396215; doi:10.1021/jacs.5c04193)
Supplement: Supplementary file 2 [file ja5c04193_si_002.zip › Data S1-S18/Description of Data S1-S18.docx]

**Supporting Data**

**Data S1:** Predicted structure of the conjugate of 5-Gly-SiP and 3G86.32 K151C, OFF state.

**Data S2:** Predicted structure of the conjugate of 5-Gly-SiP and 3G86.32 K151C, ON state.

**Data S3:** Predicted structure of the conjugate of 5-Gly-SiP and 3G86.32 F152C, OFF state.

**Data S4:** Predicted structure of the conjugate of 5-Gly-SiP and 3G86.32 F152C, ON state.

**Data S5:** Predicted structure of the conjugate of 5-Gly-SiP and 3G86.32 G153C, OFF state.

**Data S6:** Predicted structure of the conjugate of 5-Gly-SiP and 3G86.32 G153C, ON state.

**Data S7:** Predicted structure of the conjugate of 5-Gly-SiP and 3G86.32 K151C, OFF state with GFP.

**Data S8:** Predicted structure of the conjugate of 5-Gly-SiP and 3G86.32 K151C, ON state with GFP.

**Data S9:** Predicted structure of the conjugate of 5-Gly-SiP and 3G86.32 F152C, OFF state with GFP.

**Data S10:** Predicted structure of the conjugate of 5-Gly-SiP and 3G86.32 F152C, ON state with GFP.

**Data S11:** Predicted structure of the conjugate of 5-Gly-SiP and 3G86.32 G153C, OFF state with GFP.

**Data S12:** Predicted structure of the conjugate of 5-Gly-SiP and 3G86.32 G153C, ON state with GFP.

**Data S13:** Full DNA sequence of pRK393 (encoding firefly luciferase in pSBbi-GH, used for mixed co-culture to label HEK293T cells)

**Data S14:** Full DNA sequence of pMN37 (encoding DARPin Ec1 with His-tag and sortase recognition site for always-on EpCAM probe)

**Data S15:** Full DNA sequence of pMN43 (encoding DARPin 3G86.32 C76N with His-tag and sortase recognition site for always-on GFP probe)

**Data S16:** Full DNA sequence of pMN50 (encoding DARPin 3G86.32 F152C with His-tag and sortase recognition site for activatable GFP probe)

**Data S17:** Full DNA sequence of pMN211 (encoding DARPin Ec1 K152C with His-tag and sortase recognition site for activatable EpCAM probe)

**Data S18:** Full DNA sequence of pMN227 (encoding EpCAM-EGFP)
